# Engineering ultra-strong electron-phonon coupling and nonclassical electron transport in crystalline gold with nanoscale interfaces

**DOI:** 10.1038/s41467-024-55435-z

**Published:** 2025-01-02

**Authors:** Shreya Kumbhakar, Tuhin Kumar Maji, Binita Tongbram, Shinjan Mandal, Shri Hari Soundararaj, Banashree Debnath, Phanindra Sai T, Manish Jain, H. R. Krishnamurthy, Anshu Pandey, Arindam Ghosh

**Affiliations:** 1 Department of Physics, Indian Institute of Science, Bangalore, India; 2 Materials Science and Engineering, University of California Riverside, Riverside, CA USA; 3 International Centre for Theoretical Sciences, Tata Institute of Fundamental Research, Bangalore, India; 4 Solid State and Structural Chemistry Unit, Indian Institute of Science, Bangalore, India

**Keywords:** Electronic properties and materials, Nanoparticles

## Abstract

Electrical resistivity in good metals, particularly noble metals such as gold (Au), silver (Ag), or copper, increases linearly with temperature (*T*) for *T* > Θ_*D*_, where Θ_*D*_ is the Debye temperature. This is because the coupling (*λ*) between the electrons and the lattice vibrations, or phonons, in these metals is weak, with *λ* ~ 0.1−0.2. In this work, we outline a nanostructuring strategy of crystalline Au where this concept of metallic transport breaks down. We show that by embedding a distributed network of ultra-small Ag nanoparticles (AgNPs) of radius ~ 1–2 nm inside a crystalline Au shell, the electron-phonon interaction can be enhanced, with an effective *λ* as high as  ≈ 20. With increasing AgNP density, the electrical resistivity deviates from *T*-linearity and approaches a saturation to the Mott-Ioffe-Regel scale *ρ*_MIR_ ~ *ha*/*e*^2^ for both disorder (*T* → 0) and phonon (*T* ≫ Θ_*D*_)-dependent components of resistivity (here, *a* = 0.3 nm, is the lattice constant of Au).

## Abstract (web summary)

The electron-phonon interaction in gold and silver is weak, which leads to both their high conductivity and lack of conventional superconductivity. Here, Kumbhakar and coauthors find, using point contact spectroscopy, that the electron-phonon interaction in a nanostructured gold-silver film can be enhanced by over two orders of magnitude compared to the constituent elements.

## Introduction

The phenomenon of resistivity saturation in disordered metals, especially transition metals and compounds that are usually good superconductors at low temperatures, has remained an open problem in solid-state physics for over 50 years^1–9^. It is commonly agreed that saturation occurs when the scattering of electrons by either defects or thermal excitations (phonons) shrinks the mean free path to the order of inter-atomic spacing - the so-called Mott-Ioffe-Regel (MIR) limit^4,10^. The quasi-particles become incoherent, resulting in a class of ‘bad metals’. Although a comprehensive theoretical understanding of the saturation remains elusive^6–9^, the electron-phonon coupling (EPC) seems to play a key role, and resistivity saturation has been linked to, for example, the breakdown of Born-Oppenheimer approximation and delocalization of electronic states^11^, intermediate coupling of phonons to local electronic levels or hopping integrals^6,7^ or phonon-driven parallel channels of electrical conduction^8,9^. In fact, the limit of extremely strong EPC remains poorly understood in the context of metallic transport in general, at least experimentally, and it is not clear though whether the scattering rate of electrons would be limited by, for example, possible universal (Planckian) bound^12,13^, polaronic deformation^9,14^, or indeed, the stability of the metallic state itself against polaronic self-trapping^15^. The lack of understanding is partly caused by the fact that in most naturally occurring or synthesized metallic solids/alloys so far, the EPC parameter (*λ*) has not been found to exceed ~2^16^, let alone a systematic tunability of *λ* over a broad range in the same system. Controlled incorporation of disorder has been shown to assist saturation of resistivity at high temperatures, leading to the so-called ‘Mooij correlation’^17^, but the EPC remains largely intrinsic and  ≲ 1, and the fate of metallic transport in the limit *λ* ≫ 1 remains experimentally unknown.

Well-known methods to engineer EPC in solids often depend on confinement or localization of both electrons and phonons, enabled by defects, topological disorder or interfaces that cause acoustic impedance mismatch, lifting of structural symmetry, etc^18–27^. In semiconductor quantum dots and wells, Fröhlich interaction between the charge and the electric field from the optical phonons is naturally enhanced when the chemical bonds are polar in nature^28^, but placing an interface, for example, an antiphase boundary, was shown to increase the Huang-Rhys factor (a measure of the EPC in optically excited semiconductors) by orders of magnitude even in nominally weakly polar III-V crystals^18^. In metals, however, attempts to increase EPC by confining electrons^19^, confining phonons^20^, interfacial charge transfer^21^, enhanced electron surface scattering^22,29^, optical driving^23^, or application of stress^24–27^ resulted only in a moderate increase in *λ* within a factor of  ~ two. An alternate strategy involves core@shell (e.g., Au@Ag or Au@Ag@Pt) nanostructures, where the effective EPC can be continuously tuned with core/shell mass fraction owing to sound velocity mismatch at the hetero-interface^30^. Charge scattering at such heterointerfaces seems to determine the residual resistivity at low temperatures (*T*) in Ag@Au core@shell nanostructures^31^, but their effect on the EPC has not been investigated.

In this work, we have investigated EPC in noble metal hybrids consisting of a network of nanometre-sized Ag cores embedded in a crystalline Au matrix. The small intrinsic EPC and near-identical lattice constants of Ag and Au that preserve a global translation symmetry, provide a simple platform for analyzing the metallic state resistivity. Using electrical transport and point contact spectroscopy, we find that both static disorder and the EPC increase dramatically with increasing density of Ag nanoparticles (AgNP, core), *i.e*., the proliferation of buried Ag-Au interfaces. At intermediate volume fractions of Ag, *λ* as high as  ~20 could be observed, over ten times that of any known metal. This regime is also associated with a strong saturation in electrical resistivity that could be monitored by varying the EPC over nearly two orders of magnitude for the first time.

**Fig. 1:**
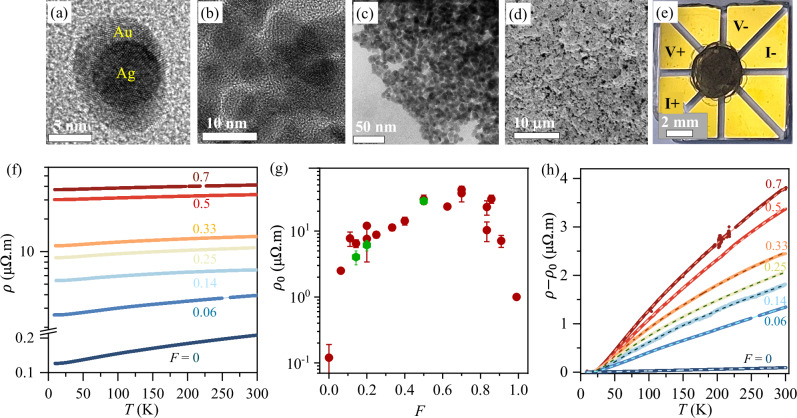
Structure and electrical transport. **a**–**e** Ag@Au nanohybrids at different length scales. **a** High-Resolution Transmission Electron Microscopy (HRTEM) image of a single AgNP of high crystallinity is embedded inside a crystalline matrix of Au. The AgNPs are typically spherical, and the interface between AgNPs and the Au host is sharp even at the atomic scale. **b** The interface sharpness remains robust even after forming a dense AgNP network within Au. **c** A snapshot of the intermediate stage of fusing (or cross-linking) of the Ag@Au nanohybrids that eventually form a continuous and compact network upon multiple loading via drop cast. **d** Scanning Electron Microscope (SEM) image of an Ag@Au film after multiple (about ten) iterations of dropcast and cross-linking. **e** Optical image of a typical Ag@Au film fabricated on pre-patterned Van der Pauw leads. *I* + , *I* − and *V* + , *V* − represent the current and volatge contacts for four-probe resistivity (*ρ*) measurements, respectively. **f** Variation of *ρ* with temperature (*T*) for films with different Ag volume fraction *F* (equivalent to AgNP density), showing metallic transport down to the lowest temperature. **g** Residual resistivity (*ρ*_0_), defined as the value of *ρ* observed at *T* ~ 6 K as a function of *F*. The red and green points were evaluated from films in Van der Pauw and Hall bar geometries, respectively. (See Fig. S6 in Supplementary Information). Error bars represent the standard deviation of the channel-to-channel statistics of resistivity in the same film. **h** Variation of the *T*-dependent component of *ρ*, obtained by subtracting *ρ*_0_ from *ρ*, reveals the emergence of sub-linear behaviour in *ρ* at high temperatures with increasing *F*. The pure AuNP film is represented by *F* = 0. Dashed lines represent fit to the data using the two-component parallel channel model given by Eq. [(2)].

Figure 1a–d show the high-resolution transmission electron microscopy (HRTEM, Fig. 1a–b; TEM Fig. 1c) and scanning electron microscopy (SEM, Fig. 1d) image of the hybrid at increasing length scales. The building block consists of solution-processed ~20−30 nm Au shells, each of which encloses multiple AgNPs of ~2 to 5 nm diameter (Fig. 1a,b). The shells are subsequently fused, or ‘cross-linked’, and compacted to form macroscopic films on a glass substrate with pre-patterned electrical leads (Fig. 1e, Fig. S6 in Supplementary Information). The Methods and Supplementary Information sections I–III describe the chemical synthesis, characterization, and film-making processes in detail. Figure 1a (and also Fig. S3 in Supplementary Information) emphasizes the sharp interface between AgNPs and the Au shell, which was found to be the case irrespective of the density $(\approx F/{r}_{{{\rm{Ag}}}}^{3})$ of the AgNPs (here, *r*_Ag_ and *F* = *V*_Ag_/(*V*_Ag_ + *V*_Au_) are the radius of the AgNP and the net relative volume fraction of Ag in the hybrid, respectively). Typically, we synthesize AgNPs of *r*_Ag_ ≈ 1−2 nm and vary *F* to tune the concentration of AgNPs, and thereby the inter-AgNP distance, *d*_Ag_ = 2*r*_Ag_/*F*^1/3^, and overall interface density, *F*/*r*_Ag_.

Figure 1f shows the *T*-dependence of the electrical resistivity (*ρ*) for Ag@Au nanohybrid films of different *F* between 6 K and 300 K. All the films showed metallic behaviour where *ρ* decreases monotonically with decreasing *T* with very little or no evidence of upturn even at *T* ~ 0.3 K (Fig. S7 in Supplementary Information). Thorough compaction and crosslinking result in geometric uniformity and electrical homogeneity better than  ~20% (Fig. S6 in Supplementary Information) and low background resistivity *ρ* ~0.2 *μ*Ω. m obtained in identically prepared films of bare Au nanoparticle (*i.e*., *F* = 0). The incorporation of AgNPs causes *ρ* to increase rapidly, which decreases again when the composite becomes Ag-rich (*F* → 1). This is seen in the variation in the residual resistivity *ρ*_0_ (defined as *ρ* at *T* ≈ 6 K) with *F*, shown in Fig. 1g. Remarkably, in the intermediate range of *F* ~ 0.4−0.8, *ρ*_0_ is nearly constant and pinned to the magnitude of ~30−40 *μ*Ω. m, which is scale of the Mott-Ioffe-Regel limit^10^, *ρ*_MIR_ = 3*π*^2^*ℏa*/*e*^2^ ≈ 10 *μ*Ω. *m* of metallic resistance for Au (*a* = 0.3 nm, is the lattice constant). At low *F* (≤0.4), *ρ*_0_ increases linearly with the overall Ag-Au interface per unit volume, suggesting that the scattering of the electrons occurs dominantly at the buried Ag-Au interfaces (Fig. S8 in Supplementary Information)^31^.

The *T*-dependent component of electrical resistivity, *i.e*., *ρ* − *ρ*_0_, separately shown in Fig. 1h, contains two important features. First, the increase in the overall magnitude of *ρ* − *ρ*_0_ with increasing *F* at any given *T*, implies increasing contribution from phonons to resistivity, and thus enhancement in the ‘effective’ EPC. Secondly, the incorporation of the AgNPs also makes the *T*-dependence of *ρ* increasingly sublinear at high temperatures for *T* > Θ_D_, where Θ_D_ ~ 150 K is the Debye temperature of Au. The coexistence of sub-linear *T*-dependence of *ρ* in disordered metals with large EPC, such as the A15 compounds, has been known for many years^2,4^, but it is not expected in crystalline noble metals such as Au or Ag (or their alloys). The sublinearity makes the Bloch-Grüneisen formula (*ρ*_BG_(*T*)) for metallic resistivity, which derives *ρ* ∝ *T* for *T* > Θ_*D*_ by treating the EPC perturbatively, evidently inadequate except for *F* = 0 and 1 (Fig. S10 in Supplementary Information). This necessitates an alternative experimental tool to quantify the EPC parameter *λ* in this case.

**Fig. 2:**
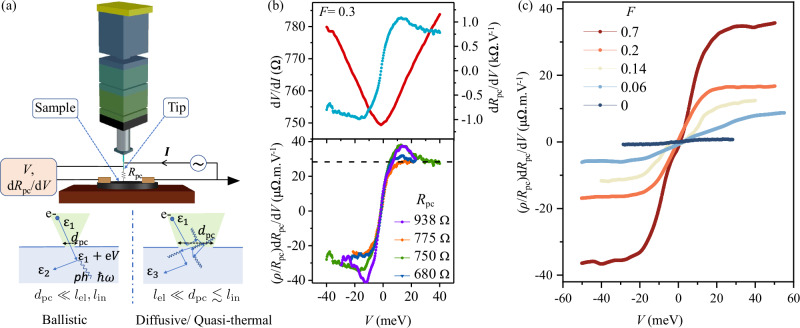
Point contact spectroscopy of Ag@Au hybrid films. **a** Schematic and processes: The tip is biased with a voltage (*V*) while the sample is grounded thereby driving current (*I*) across the tip-sample nanocontact of dimension *d*_pc_ and resistance *R*_pc_. An electron with initial energy *ϵ*_1_ gains an additional energy *eV* while passing from the tip to the sample for a ballistic contact where *l*_in_ ≫ *d*_pc_, *l*_in_ being the inelastic mean free path of the electrons. Straight lines represent electrons, while curly lines indicate the emission of phonons responsible for the dissipation of the excess energy. The dissipation of this excess energy by scattering in a ballistic contact leads to non-linearities in *I* − *V* characteristics, which can be shown to correspond to the electron-phonon interaction (EPI) function. In the case of a quasi-thermal contact, since ${l}_{{\rm{el}}},\sqrt{{l}_{{\rm{el}}}{l}_{{\rm{in}}}/3}\ll {d}_{{{\rm{pc}}}}\lesssim {l}_{{\mathrm{in}}}$ the energy of the electrons is dissipated at the orifice via inelastic collisions generating non-equilibrium phonons (*l*_el_ is the elastic mean free path). In such cases, the point contact spectrum at *eV* ≫ *ℏω*_*D*_, is a cumulative contribution from the phonons available at all energies below *ℏω*_*D*_. (See Supplementary Information Section VII for a detailed discussion.) **b** Top panel shows the bias dependence of measured d*V*/d*I* (*i.e*., *R*_pc_) and d*R*_pc_/d*V* for a typical film with Ag volume fraction *F* = 0.3. The bottom panel shows the bias dependence of normalized point contact spectrum *i.e*. (*ρ*/*R*_pc_)d*R*_pc_/d*V* for different point contact resistances which converge to a geometry-independent background (black dashed line). **c** Bias dependence of (*ρ*/*R*_pc_)d*R*_pc_/d*V* for films with different *F* measured at *T* ≈ 5 K demonstrating an increasing background value with increasing *F*. For the pure AuNP film (*F* = 0), we have plotted *d*_pc_d*R*_pc_/d*V* as discussed in Supplementary Information Section VII.D.

To estimate *λ* independently, we have performed point contact spectroscopy on the Ag@Au nanohybrid films. Figure 2a schematically explains the experimental arrangement and the underlying physical processes. In order to determine the dominant transport mechanism at the point contact, we estimated the relative spatial scales of the point contact *d*_pc_ ( ≈ 10 − 30 nm), as well as the elastic (*l*_el_ ~1 nm) and inelastic (*l*_in_~20 nm) lengths, where the latter was estimated from quantum transport measurements (Supplementary Information Section VII). We find ${l}_{{{\rm{el}}}},\sqrt{{l}_{{{\rm{el}}}}{l}_{{{\rm{in}}}}}\, \ll \, {d}_{{{\rm{pc}}}}\, \lesssim \, {l}_{{{\rm{in}}}}$, suggesting a regime intermediate to the diffusive and thermal transport, where the inelastic scattering events at the orifice can stimulate the generation of non-equilibrium phonons, which get trapped and increase the local temperature^32,33^. In this *quasi-thermal* regime, the spectral information is lost, but the cumulative effect of phonons at all available energies below *ℏω*_*D*_ causes a finite energy-independent ‘background’ that is directly proportional to the EPC. (See Methods, and Supplementary Information Section VII for more detail). *λ* is then quantitatively estimated from the energy-derivative of the resistance *R*_pc_ of a nanoscale contact between the film and a metallic Pt/Rh tip as (see derivation in Methods)^33^,

1 $$\lambda \approx \frac{3\pi ne\hslash }{16m}{d}_{{{\rm{pc}}}}{\left[\frac{{{\rm{d}}}{R}_{{{\rm{pc}}}}}{{{\rm{d}}}V}\right]}_{V\to \infty }$$

where *V*, *n* and *m* are the tip-sample bias, electron density in Au and electronic mass, respectively, and *d*_pc_ = *ρ*/*R*_pc_ is the contact diameter (Maxwell regime). The upper panel of Fig. 2b shows typical *V*-dependence of *R*_pc_ and d*R*_pc_/d*V*. At large *V*, *i.e*. *e*∣*V*∣ ≫ *ϵ*_*t*_, where *ϵ*_t_ ~ *k*_B_Θ_D_ ~ 10 − 20 meV is the energy scale beyond which the Migdal-Eliashberg function (Eq. (12)) ${\alpha }^{2}{{\mathcal{F}}}(\omega )\to 0,[{d}_{{{\rm{pc}}}}{{\rm{d}}}{R}_{{{\rm{pc}}}}/{{\rm{d}}}V]$ becomes independent of the geometric details of the contact, as seen from the convergence of the traces at different *R*_pc_ (lower panel of Fig. 2b). Figure 2c shows the *V*-dependence of *d*_pc_d*R*_pc_/*dV* for different values of *F*. The rapid increase in its large-*V* magnitude with increasing *F* confirms the enhancement in the EPC with the incorporation of AgNPs.

**Fig. 3:**
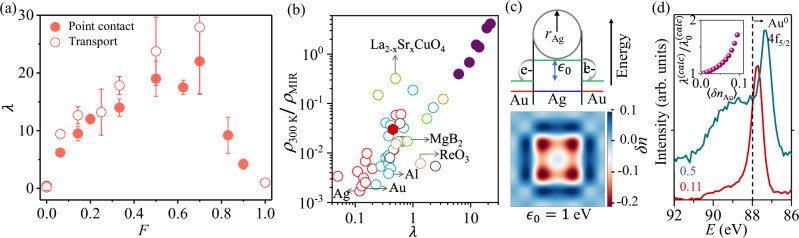
Electron-phonon coupling in Ag@Au hybrids. **a** Electron-phonon coupling constant (*λ*) estimated from point contact measurements and electrical transport (*ρ* − *T*) data as function of Ag filling *F*. Error bars in the open and filled points, representing *λ* have been estimated from the error in resistivity, *ρ*, shown in Fig. 1g by error propagation. **b** Room temperature resistivity (*ρ*_300 K_) for different materials is normalized by the respective Mott-Ioffe-Regel resistivity (*ρ*_MIR_) and plotted as a function of electron-phonon coupling constant, *λ*. Red, blue, green, and yellow open circles represent non-superconducting metals, metals/alloys that superconduct at low *T*, intermetallic compounds, and high-*T*_*c*_ cuprates, respectively (see Supplementary Information VIII for detail). Filled red and purple circles represent films of pure Au nanoparticle and Ag@Au hybrids for different *F* values, respectively. **c** (Top Panel): A schematic of the electrochemical potential of electrons at the Ag and Au sites across the Ag@Au nanohybrid, *ϵ*_0_ being the potential difference between them. Electrons transfer from a higher onsite potential at Ag to a lower potential in Au. (Bottom Panel): Theoretical computation of the excess electron occupancy, *δn* in a square lattice toy model (See Methods and Supplementary Information Section IX), where Ag is embedded inside Au. **d** Binding energy peak of the Au-4*f*_5/2_ peak from X-ray photoelectron spectroscopy (XPS) is shown for two different values of *F* = 0.11, 0.5. The data is shifted vertically for clarity. (See Fig. S5 and Section II.B in Supplementary Information for more details). The dotted line represents the binding energy peak of Au-4*f*_5/2_ core-level of Au^0^. Inset shows the theoretically computed EPC on the square lattice toy model, *λ*^(calc)^ with the average excess electron occupancy (〈*δn*_Au_〉) on Au.

Figure 3 a shows the magnitude of *λ*, obtained from Eq. (1) using *d*_pc_d*R*_pc_/d*V* values at large *V*, as function of *F*. Remarkably, we find *λ* can be as high as  ≈ 20 for *F* ~ 0.5 − 0.7, before dropping to ~1, expected for a film of small AgNPs^29^. Both the magnitude and the *F*-dependence of *λ* from point contact spectroscopy are consistent within 20% with the estimates obtained by fitting the *ρ* –*T* data with the Bloch-Grüneisen formula at low *T* (≤100 K) (open symbols in Fig. 3a, see Methods and Fig. S10 in Supplementary Information for details). Such large *λ* is unprecedented in metallic solids and exceeds those with strong EPC, for example, the A15 compounds, by at least a factor of  ~ ten (Fig. 3b, also see Supplementary Information section VIII for details). Intriguingly, the extended correlation between *λ* and the normalized resistivity in Fig. 3b suggests Ag@Au hybrids to be an ‘extreme’ case of a metal where the electron-phonon scattering drives *ρ* → *ρ*_MIR_ even at room temperature.

Surface scattering can increase EPC in nanostructured metal films compared to bulk^22^, but such enhancements are within a factor of  ~ two, and thus much smaller than the enhancement in *λ* observed here. The formation of the solution-processed Ag@Au core-shell nanoparticle hybrids and their stability against galvanic replacement is critically dependent on the interfacial charge transfer that results in the formation of electric dipoles across the hetero-interface^34–37^. The charge transfer can be parametrized from the difference in on-site energies (*ϵ*_0_) of Au and Ag, which is distributed as on-site excess charge *δn* (bottom panel, Fig. 3c), and illustrated for a 4 × 4 Ag atom array embedded in an array of Au atoms (details in Supplementary Information Section IX). We experimentally verified such transfer of charge in our nanohybrids using X-ray photoelectron spectroscopy (XPS), where a red-shift in the Au (4*f*_5/2_) with respect to neutral Au^0^ (4*f*_5/2_) suggests negative charge doping in Au (Fig. 3d). More detailed XPS results are available in Fig. S5 in Supplementary Information, which indicates average electron doping of the Au atoms by as much as ~0.6 ± 0.1 per atom for *F* = 0.5 (Supplementary Information Section II.B). The radial dipoles across the interface, formed when the Ag^∣*δn*∣+^ and Au^∣*δn*∣−^ sites assume opposite oxidation states can couple strongly to the lattice phonons via long-range Coulomb interactions. A detailed analytical and computational model on the two-dimensional array of Ag@Au hybrid indeed confirms additional contributions to the electron-phonon matrix elements, *g* through the inter-site Coulomb interaction, thereby enhancing the Migdal-Eliashberg function ${\alpha }^{2}{{\mathcal{F}}}(\omega )$ [Eq. (12)], and thus *λ*^38^ (see inset of Fig. 3d, Methods and Supplementary Information Section IX).

**Fig. 4:**
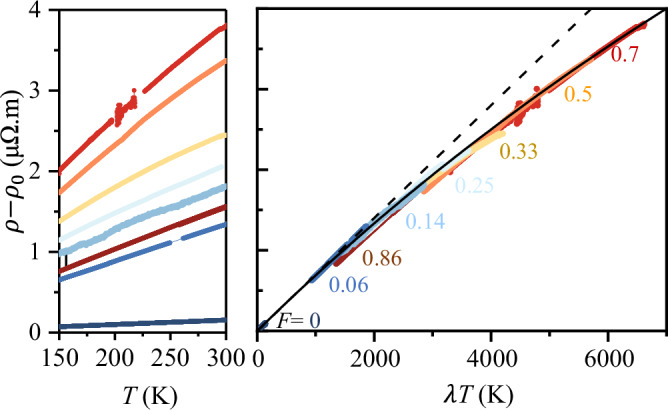
Scaling of resisitivity and electron-phonon coupling strength. Universality in the *T*-dependent component of *ρ* for *T* > Θ_D_~150 K (left panel) for all *F* with *T* scaled with the corresponding EPC parameter *λ* estimated from point contact spectroscopy measurements (right panel). The dashed line in the right panel indicates the ‘Planckian’ resistivity (=2*mπk*_B_*λT*/(*ℏne*^2^)). The solid line is a fit to (*ρ* − *ρ*_0_) according to the two-component parallel channel model given by Eq. [(2)] (see text).

We now focus on the resistivity saturation at *T* ≫ Θ_D_, which probably is the ‘smoking gun’ signature of the strong emergent EPC in Ag@Au hybrids. In fact, our ability to vary *λ* by over a factor ~200 (from bare gold film to Ag@Au nanohybrid at *F* ≈ 0.7), allows access to the phonon contribution to resistivity dynamically from weak to ultra-strong coupling regime on a single material platform for the first time. In Fig. 4, we plotted the high-temperature segment (300 K ≥*T* ≥ 150 K, i.e., *T* ≥ Θ_D_) of (*ρ* − *ρ*_0_) shown in Fig. 1h for all *F*, where the temperature axis is scaled by the corresponding *λ*, obtained from the point contact measurements. Two key observations can be summarized as follows: First, the collapse of the resistivity traces for different *F* onto a single one suggests *λT* would continue to be the ‘scaling variable’ that determines the resistivity even at very large *λ*, although the perturbative limit with linear scattering rate  ≈ 2*πk*_B_*λT*/*ℏ*, is expectedly recovered only when *λ* → 0 (dashed line). Second, the sublinearity in (*ρ* − *ρ*_0_) at large *λT*, representing ‘resistivity saturation’, can be modelled with

2 $$\frac{1}{\rho -{\rho }_{0}}=\frac{1}{{\rho }_{{{\rm{BG}}}}}+\frac{1}{{\rho }_{| | }}$$

which resembles a “parallel resistor channel” with *ρ*_BG_(*T* ≥ Θ_D_) = 2*πmk*_B_*λT*/(ℏ*ne*^2^)^16,39^ (See Methods and Supplementary Information V for details), and *ρ*_∣∣_ is the resistivity of a parallel non-classical channel whose universality, temperature dependence, or even existence, have been questioned many times, but without a satisfactory answer so far^2,4,8^. The solid line fit in Fig. 4 corresponds to *ρ*_∣∣_ ≈ 20 *μ*Ω. m implying that the phonon contribution to resistivity can be described by an ‘ideal’ Bloch-Grüneisen behaviour in parallel to a *T*-independent non-classical channel of resistivity close to the MIR-limit. In fact, the variation in *ρ*(*T*) over the entire experimental temperature range can be satisfactorily captured by using the full form of *ρ*_BG_(*T*) and Eq. (2) (dashed lines in Fig. 1h). See Methods and Supplementary Information Section V for further discussions on the parallel resistor formula and other fit protocols.

Our experiment confirms the long-suspected inevitability of resistivity saturation in metals^3^, irrespective of the strength of the EPC. However, there are deeper consequences. The first concerns the question, is there a universal bound to the EPC for a metal to exist? The persistence of metallic transport in Ag@Au hybrids with *λ* ≫ 1, is *prima facie* at odds with a stability bound observed in Monte Carlo calculations on the Holstein model^15^, or other fundamental ‘Planckian limits’ to dissipation or thermalization in metals^12,13^. An important consideration would, however, be the heterogeneous nature of our system, where the electrons couple to engineered vibration modes of a foreign species, such as the surface phonon modes of AgNPs. In this aspect, our system is fundamentally different from homogenous crystalline metals, which are unstable towards the formation of polaronic insulators at strong EPC. Nonetheless, the *T*-dependence of *ρ* can also be fitted with a thermally activated parallel conduction channel (See Section V in Supplementary Information), and thus a possible coexistence of polaronic insulating phase and itinerant electrons cannot be ruled out^40–42^. Second, there is also a discrepancy with the models of resistivity saturation built on the breakdown of Born-Oppenheimer approximation and Matthiessen’s rule, for example, polaronic deformation of disorder^43^ or phonon-assisted delocalization^8,9,11^. These mechanisms often involve crossover to negative temperature coefficient of resistivity at *ρ* ~ *ρ*_MIR_, i.e., ‘Mooij correlation’^17,43^, which was not observed in any of our samples. The absence of Anderson localization itself, even for the very strong disorder at *F* ~ 0.5 − 0.8 at low *T* (down to ~0.3 K, See Fig. S7 in Supplementary Information), indicates that, unlike a conventional disordered metallic phase, the phase-coherent effects here are also modified. Finally, we also consider the role of long-range electron-electron interaction, which can impact transport in multiple ways, including assisting in the delocalization of carriers^44^, introducing hydrodynamic viscosity^45^, or even the suppression of resistivity saturation itself^2,4,5^. Such interaction is expected to be weak at metallic densities (measured by the low-field Hall effect (Fig. S9 in Supplementary Information)), although a modification of this scenario due to the presence of many-body effects driven by phonons at the buried interfaces cannot be ruled out^46^.

In conclusion, we have reported the realization of a metallic hybrid composed of ultra-small silver cores dispersed in a crystalline gold matrix, in which the electrical resistivity shows saturation as the silver core density and temperature are increased. Both electrical transport and point contact spectroscopy reveal that the electron-phonon coupling *λ* in these engineered metallic hybrids can be as large as ~20, more than ten times than any known metallic solid. Our experiments outline a novel strategy to modify some of the fundamental properties of solids utilizing buried interfaces at the nanoscale.

## Methods

### Chemical synthesis

Ag@Au nanohybrids (Ag@Au NHs) were synthesized using a colloidal approach^31,47^. The synthesis process involved two sequential stages: the reduction of AgNO_3_ with ice-cold NaBH_4_ to form Ag nanoparticles (AgNPs) in an aqueous solution (in ultrapure water, purity~18.2 MΩ.cm) containing NaOH, NH_4_Br, KI, and CTAB as a capping agent, followed by the introduction of HAuCl_4_ at 40^∘^C with continuous stirring. In situ, UV-Vis absorption spectroscopy was employed to monitor the synthesis process, and the reaction was terminated by adding isopropyl alcohol (IPA), resulting in nanohybrid agglomeration. The solution was then centrifuged at 10, 000 rpm for 15 minutes to remove excess CTAB and isolate the nanohybrids.

### Characterization

#### UV-Vis Spectroscopy

UV-vis absorption spectroscopy was used to assess the formation of AgNPs and Ag@Ag nanohybrids. The addition of NaBH_4_ in the reaction mixture containing AgNO_3_, at a specific ’wait time’ (*t*) resulted in a prominent peak around  ~393 nm, corresponding to the localized surface plasmon resonance (LSPR) absorption band of ultra-small AgNPs. Upon introducing HAuCl_4_, the LSPR peak underwent a redshift to  ~524 nm over approximately 1000 seconds, indicating the formation of a thicker shell due to spontaneous interdiffusion. To ensure a well-defined AgNP surface, we employed a strategic approach. Monitoring the SPR band shift with UV-Vis spectroscopy, we terminated the reaction by adding isopropyl alcohol (IPA) approximately 30 seconds after HAuCl_4_ addition.

#### X-Ray Photo-electron Spectroscopy

An Axis Ultra K*α* X-ray photoelectron spectrometer with a monochromatized photon energy of  ~1486.6 eV was used for all XPS measurements. To minimize moisture absorption, the sample was quickly inserted into the load-lock of the instrument, pumped in the entry chamber until the pressure around 10^−8^ mbar was reached, and subsequently transferred to the analysis chamber. The individual core-level spectra were corrected for charging using C-1*s* peak at 284.5 eV as standard. The peak fitting of the individual core-levels was done using Casa XPS software.

#### Transmission Electron Microscopy

Transmission Electron Microscopy (TEM) was employed to examine the structural characteristics of an Ag@Au nanohybrid. The FEI TITAN Themis TEM operating at 300 kV, which offers a point resolution of  ~0.2 nm and an energy spread of 0.136 nm, was utilized to capture all TEM images. High-Resolution Transmission Electron Microscopy (HRTEM) images were obtained along the zone axis to assess the Ag-Au interface and investigate any defects that may have arisen within the bimetallic entities. To prepare the sample for TEM imaging, the nanoparticle underwent multiple cleaning cycles in a chloroform (CHCl_3_) : methanol mixture (1: 3 ratio), followed by centrifugation at 15000 rpm for 5 minutes. The resulting precipitated sample was then dispersed in chloroform and deposited onto a carbon-coated TEM grid, which was subsequently dried under vacuum overnight.

### Film preparation

The drop-cast technique was employed to fabricate the Ag@Au NH film onto prepatterned Cr/Au contacts (with thickness of  ≈ 10 nm/60 nm) arranged in various lead configurations on a glass substrate. Prior to drop-casting onto the pre-patterned leads, the sample was dissolved in CHCl_3_. Subsequently, the sample was dried at 70 °C for 30 seconds and washed with deionized (DI) water followed by KOH solution and IPA to eliminate any excess CTAB and achieve a chemically sintered cross-linked nanostructure. This process was repeated ten times for each film, resulting in an average film thickness of *t*_f_ ≈ 3 ± 0.5 *μ*m and a diameter of  ≈ 4 mm, typically covering the leads (Fig. 1e of Main Manuscript, Fig. S6 in Supplementary Information).

### Electrical Measurement

Four-probe resistivity of the sample was measured down to temperature (*T*) ~6 K in a home-built cryostat by passing a DC current of  ~100 *μ*A with Keithley 6221 and measuring the voltage with Keithley 2182A. The voltages across multiple contacts were recorded using the Keithley 3700 Multiplexer card. The voltage was measured in delta mode to cancel any thermo-emf across the contacts. Resistivity from *T* ~10 K down to *T*~0.3 K was measured in a He3 cryostat.

### Fitting of *ρ* − *T* data

The resistivity (*ρ*) of metal with electron-phonon interaction playing the dominant role of scattering can be expressed in terms of the Bloch Grüneisen^16,39^ form as:

3 $$\rho (T)={\rho }_{0}+{\rho }_{{{\rm{BG}}}}(T)$$

where *ρ*_0_ is the residual resistivity, and

4 $${\rho }_{{{\rm{BG}}}}=\frac{2\pi \lambda {k}_{{{\rm{B}}}}/{{{\Theta }}}_{{{\rm{D}}}}}{(n/m){e}^{2}}{\left(\frac{T}{{{{\Theta }}}_{{{\rm{D}}}}}\right)}^{5}\int_{\!\!\!\!0}^{{{\Theta }}_{{{\rm{D}}}}/T}\frac{{x}^{5}}{({e}^{x}-1)(1-{e}^{-x})}dx$$

is the Bloch Grüneisen form of resistivity arising from electron-phonon scattering. Θ_D_, the Debye temperature, and *λ*, the electron-phonon coupling constant can be estimated by fitting the *ρ* − *T* data with Eq. [(3)]. As shown in Fig. 1h and Fig. S10 in Supplementary Information, we have fitted the *ρ* − *T* data of AuNP and AgNP films using Eq. [(3)]. Θ_D_ ~ 170 K, and *λ* ~ 0.45 for Au, and Θ_D_ ~ 190 K and *λ* ~ 1 for Ag are estimated as fit parameters. The increased value of *λ* as compared to the bulk value of ~0.2 for Au and Ag could be attributed to the nanostructuring in the film and increased electron scattering from the surfaces^22^. For Ag@Au films, Eq. [(3)] cannot describe *ρ* − *T* for the entire range of *T*. Fig. S10 in Supplementary Information shows that the transport data for *F* = 0.5 deviates from the low-temperature BG fit (*T* ≤ 100 K) to the data. For fitting the *ρ* − *T* data for Ag@Au hybrid films, Eq. [2] is used where *ρ*_∥_, Θ_D_ and *λ* are the parameters of fit. Θ_D_ obtained from fitting *ρ* − *T* of Ag@Au films with Eq. [(3)] in the low *T* range, and with Eq. [2] with a parallel conduction channel, are consistent and lies within the range of 150 − 170 K for all films (Fig. S10 in Supplementary Information). Θ_D_ being close to the Debye temperature of Au (Θ_D,Au_ ~ 170 K) in all cases indicates the electrical conduction occurs primarily within the host lattice of Au. *λ* derived from the low-*T* Bloch Grüneisen fit is shown in Fig. 3a. However, *λ* estimated from parallel channel fit is slightly overestimated (~20%) and probably less accurate since the scattering mechanism with this model, even at low *T*, is not purely electron-phonon mediated. *ρ*_∥_ estimated from the parallel channel fit, given by Eq. [2], is plotted in Fig. S10d of Supplementary Information as a function of *F* after normalizing with *ρ*_MIR_. *ρ*_∥_ ≈ 8 − 25 *μ*Ω. m is *T*-independent, and lies within a factor of two of *ρ*_MIR_ ~ 10 *μ*Ω.m, which drives the saturation of *ρ*(*T*).

### Point contact measurements

#### Experimental setup

A sharp Pt/Rh metallic tip is brought in contact with the film in a controlled manner with the help of nanopositioners (attocubes and piezo tubes) as indicated in the schematic of the experimental set-up in Fig. 2(a) of main manuscript and Fig. S12 in Supplementary Information. The tip-sample chamber is loaded inside a home-built cryostat that could be cooled down to *T* ~ 5 K.

#### Theoretical framework for analysis

Point contact measurements are done with the technique of modulation spectroscopy^33,48^ which measures the higher-order derivatives of a signal through its AC components at harmonics of a definite frequency. The circuit for measurement of the point contact voltage is shown in Fig. S12 of Supplementary Information. A mixed AC+DC current $I+{i}_{{{\rm{m}}}}\sin (\omega t)$ is passed through the sample using a constant current circuit. AC voltage from a lock-in amplifier and DC voltage from Keithley 2400 are added with an op-amp adder. A series resistance *R*_s_ ≫ *R*_pc_ is used to achieve the constant current in the circuit. The voltage across the tip-sample contact can be represented as a Taylor series expansion of the *I* − *V* curve as follows:

5 $$V=f(I+{i}_{{{\rm{m}}}}\sin (\omega t))	={\sum }_{k=1}^{\infty }\frac{{i}_{{{\rm{m}}}}^{k}}{k!}\frac{{{\rm{d}}}{V}^{k}}{{{{\rm{d}}}}^{k}I}{\sin }^{k}(\omega t)\\ 	=V+{i}_{{{\rm{m}}}}\sin (\omega t)\frac{{{\rm{d}}}V}{{{\rm{d}}}I}+\frac{1}{2!}\frac{{{{\rm{d}}}}^{2}V}{{{\rm{d}}}{I}^{2}}{i}_{{{\rm{m}}}}^{2}{\sin }^{2}(\omega t)\\ 	\quad+\frac{1}{3!}\frac{{{{\rm{d}}}}^{3}V}{{{\rm{d}}}{I}^{3}}{i}_{{{\rm{m}}}}^{3}{\sin }^{3}(\omega t)+...\\ 	=1\left(V+\frac{{i}_{{{\rm{m}}}}^{2}}{4}\frac{{{{\rm{d}}}}^{2}V}{{{\rm{d}}}{I}^{2}}+...\right)\\ 	\quad+\sin (\omega t)\left({i}_{{{\rm{m}}}}\frac{{{\rm{d}}}V}{{{\rm{d}}}I}+\frac{{i}_{{{\rm{m}}}}^{3}}{8}\frac{{{{\rm{d}}}}^{3}V}{{{\rm{d}}}{I}^{3}}+...\right)\\ 	\quad -\cos (2\omega t)\left(\frac{{i}_{{{\rm{m}}}}^{2}}{4}\frac{{{{\rm{d}}}}^{2}V}{{{\rm{d}}}{I}^{2}}+\frac{{i}_{{{\rm{m}}}}^{4}}{12}\frac{{{{\rm{d}}}}^{4}V}{{{\rm{d}}}{I}^{4}}+...\right)+..$$

Grouping terms of the same amplitude of the modulation frequency *ω* gives

6 $$V={V}_{0}+{\sum }_{i=n}^{\infty }({a}_{2n-1}\sin ((2n-1)\omega t)+{a}_{2n}\cos (2n\omega t))$$

For a small AC current *i*_m_ ≪ *I*, the higher-order terms in the series expansion, which vary as *i*^*n*^ can be neglected in each group and the amplitude of the voltage at frequency *nω* becomes proportional to the *n*-th order derivative *.i.e.* *a*_*n*_ ∝ d^*n*^*V*/d*I*^*n*^. Also, it is to be noted that the odd harmonics are in-phase (sine component) with the source AC signal ${i}_{{{\rm{m}}}}\sin (\omega t)$, and even harmonics are out of phase, at 90° (cosine component) with the signal.

Hence, the first order derivative can be estimated from the amplitude of the *ω* component at 0° phase as in Eq. [(5)].

7 $${a}_{1}	={i}_{{{\rm{m}}}}\frac{{{\rm{d}}}V}{{{\rm{d}}}I}\\ \frac{{{\rm{d}}}V}{{{\rm{d}}}I}	=\frac{{a}_{1}}{{i}_{{{\rm{m}}}}}$$

The second order derivative can be derived from the amplitude of the 2*ω* component at 90° phase as in Eq. [(5)].

8 $${a}_{2}	=-\frac{{i}_{{{\rm{m}}}}^{2}}{4}\frac{{{{\rm{d}}}}^{2}V}{{{\rm{d}}}{I}^{2}}\\ \frac{{{{\rm{d}}}}^{2}V}{{{\rm{d}}}{I}^{2}}	=-\frac{4{a}_{2}}{{i}_{{{\rm{m}}}}^{2}}$$

Both *ω* and 2*ω* components of the voltage difference across the tip-sample junction are acquired simultaneously with two lock-in amplifiers as denoted by *V*_*ω*_, and *V*_2*ω*_ in Fig. S12 of Supplementary Information respectively, after amplification with SR 560. The sample is grounded through current-voltage amplifier SR 570, which allows us to constantly monitor the current through the sample and hence tune the point contact resistance (*R*_pc_).

Since a lock-in amplifier shows the rms value of a signal, the amplitude of the signal is $\sqrt{2}$ times the measured value *i.e*. ${a}_{n}={V}_{n\omega }\sqrt{2}$, where *V*_*nω*_ is the measured signal at *nω*. If *V*_AC_ is the voltage at the sine output of the lockin amplifier, the AC current through the sample is ${i}_{{{\rm{m}}}}={V}_{{{\rm{AC}}}}\sqrt{2}$. *V*_*ω*_, being the measured voltage by the lock-in amplifier at *ω* component and 0° phase, the amplitude of the voltage drop across the tip and sample is expressed using Eq. [(7)] as

9 $$\frac{{{\rm{d}}}V}{{{\rm{d}}}I}=\frac{{a}_{1}}{{i}_{{{\rm{m}}}}}=\frac{{V}_{\omega }\sqrt{2}}{{V}_{{{\rm{AC}}}}\sqrt{2}/{R}_{{{\rm{s}}}}}=\frac{{V}_{\omega }}{{V}_{{{\rm{AC}}}}/{R}_{{{\rm{s}}}}}$$

Similarly, *V*_2*ω*_, being the signal measured by the lockin amplifier at 2*ω* component and 90° phase, the second order derivative is expressed using Eq. [(8)] as

10 $$\frac{{{{\rm{d}}}}^{2}V}{{{\rm{d}}}{I}^{2}}=-\frac{4{V}_{2\omega }\sqrt{2}}{{(\sqrt{2}{V}_{{{\rm{AC}}}}/{R}_{{{\rm{s}}}})}^{2}}=-\frac{4{V}_{2\omega }}{\sqrt{2}{({V}_{{{\rm{AC}}}}/{R}_{{{\rm{s}}}})}^{2}}$$

d*V*/d*I* corresponds to the point contact resistance *R*_pc_ and d^2^*V*/d*I*^2^ is related to the derivative of *R*_pc_ with bias as d^2^*V*/d*I*^2^ = *R*_pc_d*R*_pc_/d*V*.

With appropriate multi-stage vibration isolation, *R*_pc_ ranging from 500 Ω to 2 KΩ could be stabilized typically in the Ag@Au hybrid films with attocube and piezo controllers by monitoring the current through the sample. The modulation AC current *i*_m_ was typically fixed at 1−5 *μ*A, whereas the DC current/was varied till  ~100 −200 *μ*A in magnitude. *R*_pc_ can be expressed as a combination of ballistic Sharvin resistance (${R}_{{{\rm{sh}}}}=16\rho l/3\pi {d}_{{{\rm{pc}}}}^{2}$) and diffusive Maxwell resistance^33^ (*R*_M_ = *ρ*/*d*_pc_).

11 $${R}_{{{\rm{pc}}}}=\frac{16\rho l}{3\pi {d}_{{{\rm{pc}}}}^{2}}+\frac{\rho }{{d}_{{{\rm{pc}}}}}$$

where *d*_pc_ is the diameter of the point contact orifice as shown in the schematic of Fig. 2b of the main manuscript. For an ideal ballistic point contact, the derivative of the point contact resistance represents the Migdal Eliashberg spectral function (Eq. [(12)]) $g(\epsilon )={\alpha }^{2}{{\mathcal{F}}}(\epsilon )$^33^, given by

12 $${\alpha }^{2}{{\mathcal{F}}}(\omega )={{\mathcal{G}}}(\omega )=\propto {\sum}_{k,q}| g(k,q){| }^{2}\delta ({\varepsilon }_{k})\delta ({\varepsilon }_{k+q})\delta (\omega -{\omega }_{q})$$

where *k* is the electron wave vector, *q* and *ω*_*q*_ are the phonon wave vector and frequency, respectively; *A* is a normalization constant and *g*(*k*, *q*) are the electron-phonon matrix elements. The Migdal Eliashberg function represents the probability of specific phonon modes (with energy *ϵ*) to decay into an electron-hole pair and closely resemble the phonon density of states in most cases.

13 $$\frac{1}{{R}_{{{\rm{sh}}}}}\frac{{{\rm{d}}}{R}_{{{\rm{pc}}}}}{{{\rm{d}}}V}=\frac{8e{d}_{{{\rm{pc}}}}}{3\hslash {v}_{{{\rm{F}}}}}{{\mathcal{G}}}(\epsilon ){| }_{\epsilon=eV}$$

The integral of ${{\mathcal{G}}}(\epsilon )$ is a measure of the electron-phonon coupling constant.

14 $$\lambda=2\int_{\!\!\!\!0}^{\infty }\frac{{{\mathcal{G}}}(\epsilon )}{\epsilon }d\epsilon$$

Hence, from the measurement of the point contact spectrum, we can estimate the electron-phonon coupling parameter.

An ideal ballistic contact is not achieved in experiments, and hence, one needs to consider non-equilibrium processes to analyze the point contact spectrum. However, *λ* can be estimated from the non-zero background of the point-contact spectrum (*eV* ≫ *ℏω*_*D*_), irrespective of the nature of the contact as long as phonons are the primary source of inelastic scattering, and elastic scattering dominates over the inelastic scattering processes. These conditions are satisfied in our system. See Supplementary Information Section VII for detailed derivations.

It can be shown d*R*_PC_/d*V* multiplied by the point contact diameter, *d*_PC_ is directly proportional to *λ*, with the proportionality factor determined by the mass *m*, and number density *n* of the electrons. Specifically, we get,

15 $${d}_{{{\rm{PC}}}}{\left.\frac{{{\rm{d}}}{R}_{{{\rm{PC}}}}}{{{\rm{d}}}V}\right| }_{V\to \infty }=\frac{16f}{3\pi }\frac{m}{ne\hslash }\lambda$$

where the constant *f* ~ 1.1−1.8, for a contact varying from diffusive to thermal regimes of transport. Using a typical value of resistivity for Ag@Au nanohybrid films *ρ* ~ 10 *μ*Ω.m, we can estimate the mean free path, *l* from the Drude expression *ρ* = *mv*_*F*_/*ne*^2^*l* (ν_F_ is the Fermi velocity) as *l* ≈ 0.1 nm, which is smaller than mechanically achieved point contacts. This suggests a diffusive/thermal nature of the contact since *l* ≪ *d*_pc_. *d*_PC_ is estimated by assuming the point-contact resistance to be arising primarily from the Maxwell contribution .i.e *R*_PC_ = *ρ*/*d*_PC_. The estimation of *d*_PC_ is validated by the scaling of *ρ*/*d*_PC_ d*R*_pc_/d*V* at different values of *R*_pc_, as shown in Fig. 2c of the main manuscript. Since for the typical values of point contact that we could achieve, *d*_PC_ ≲ *l*_in_, we have used *f* ~ 1.1, which is the case for the diffusive regime of point contact transport. Eq. [(15)] can be inverted to derive *λ* as:

16 $$\lambda=\frac{3\pi }{16}\frac{ne\hslash }{m}{\left[\frac{\rho }{{R}_{{{\rm{pc}}}}}\frac{{{\rm{d}}}{R}_{{{\rm{pc}}}}}{{{\rm{d}}}V}\right]}_{V\to \infty }$$

Further details regarding the theoretical framework and analysis are discussed in Supplementary Information Section. VII

### Computational details

The theoretical calculations for the charge transfer were done on a 2D ‘toy model’ where a periodic array of (4 × 4) clusters of “Ag” sites are surrounded by 48 “Au” sites forming a superlattice of (8 × 8) 2D supercells. The calculations were carried out using the model Hamiltonian described in Supplementary Information Section IX. The relative ratio of the number of atoms for Ag:Au has been taken to be 1:3 to resemble a typical fraction *F* = 0.25 of the Ag@Au core@shell nanohybrid. Due to the mismatch of the local potential seen by the conduction electrons localized in Wannier orbitals at the Ag and Au sites, as shown in the top panel of Fig. 3c, and the long-range Coulomb interactions between electrons occupying these Wannier orbitals, there is a charge transfer at each site, indicated by the excess electron occupancy *δn*. The amount of charge transferred can be tuned to mimic the experimental values by varying the onsite potential difference between Au and Ag atoms, *ϵ*_0_.

As shown in Fig. 3c of the text, Au and Ag atoms become electron and hole-doped, respectively, for most positive values of *ϵ*_0_, which is also consistent with the X-ray photoelectron spectroscopy (XPS) data.

The parameters used in our study are as follows: the nearest neighbour distance, *d*_1_, within the square lattice considered is set to 4.10 Å, corresponding to the lattice spacing in FCC-Ag/Au. The nearest neighbour hopping between all the atomic sites is set at 1 eV, and the hopping decay factor, *ξ*_0_ = *d*_1_, is adjusted to yield the second-nearest neighbour hopping of 0.5 eV. The on-site Coulomb interaction energy is fixed at *U*_0_ = 2 eV, and *V*_0_, which controls the strength of the long-range Coulomb interactions, is chosen such that *V*_0_/*d*_1_ = 2 eV. The difference in work function between Au and Ag atoms is incorporated into the on-site potentials, with ${\epsilon }_{0}=({\epsilon }_{j}^{\,{\mbox{Ag}}}-{\epsilon }_{j}^{{\mbox{Au}}\,})$. The charge density obtained from solving the self-consistent mean field equations at $({\epsilon }_{j}^{\,{\mbox{Ag}}}-{\epsilon }_{j}^{{\mbox{Au}}\,})=1$ eV is presented in the bottom panel of Fig. 3c of the main manuscript. More detailed figures are provided in the Supplementary Information Section IX.

Phonons are computed with spring constants of 1.0 for nearest neighbour atoms and 0.5 for next-nearest neighbours in arbitrary units. Electronic and phononic band spectra are calculated on (16 × 16) **k, q** grids within the Brillouin zone of the supercell and are used to determine the electron-phonon coupling strength (*λ*^(calc)^) using the ‘double delta’ approximation. Variation of *λ*^(calc)^ with 〈*δn*_Au_〉, average electron occupancy on Au sites is shown in the inset of Fig. 3d, where 〈*δn*_Au_〉 has been tuned by changing *ϵ*_0_ from 0.1 eV to 4 eV.

## Supplementary information

Supplementary Information

Transparent Peer Review file

## Source data

Source Data

## Data Availability

Data that support the plots within this paper, and other findings of this study are available from the corresponding author upon reasonable request. Source data are provided with this paper.

## Code availability

The codes that support the findings of this study are available from the corresponding author upon reasonable request.

## References

[1] Fisk, Z. & Lawson, A. Normal state resistance behavior and superconductivity. *Solid State Commun.* **13**, 277–279 (1973).

[2] Wiesmann, H. et al. Simple model for characterizing the electrical resistivity in *a* − 15 superconductors. *Phys. Rev. Lett.* **38**, 782–785 (1977).

[3] Allen, P. B. Theory of resistivity ‘saturation’. In Suhl, H. & Maple, M. B. (eds.) *Superconductivity in D- and F-Band Metals*, 291–304 (Academic Press, 1980). https://www.sciencedirect.com/science/article/pii/B978012676150450038X.

[4] Gunnarsson, O., Calandra, M. & Han, J. Colloquium: Saturation of electrical resistivity. *Rev. Mod. Phys.***75**, 1085 (2003).10.1103/PhysRevLett.87.26660111800849

[5] Hussey, N., Takenaka, K. & Takagi, H. Universality of the mott–ioffe–regel limit in metals. *Philos. Mag.* **84**, 2847–2864 (2004).

[6] Calandra, M. & Gunnarsson, O. Saturation of electrical resistivity in metals at large temperatures. *Phys. Rev. Lett.***87**, 266601 (2001).11800849 10.1103/PhysRevLett.87.266601

[7] Millis, A. J., Mueller, R. & Shraiman, B. I. Fermi-liquid-to-polaron crossover. i. general results. *Phys. Rev. B* **54**, 5389–5404 (1996). 10.1103/physrevb.54.5389 9986498

[8] Werman, Y. & Berg, E. Mott-ioffe-regel limit and resistivity crossover in a tractable electron-phonon model. *Phys. Rev. B* **93**, 075109 (2016).

[9] Werman, Y., Kivelson, S. A. & Berg, E. Non-quasiparticle transport and resistivity saturation: a view from the large-n limit. *npj Quantum Mater.* **2**, 7 (2017).

[10] Ioffe, A. & Regel, A. Non-crystalline, amorphous, and liquid electronic semiconductors. In *Prog. Semicond.*, **4**, 237–291 (1960).

[11] Li, J. & Drabold, D. A. Electron hopping between localized states: A simulation of the finite-temperature anderson problem using density functional methods. *Phys. Rev. B* **68**, 033103 (2003).

[12] Bruin, J. A. N., Sakai, H., Perry, R. S. & Mackenzie, A. P. Similarity of scattering rates in metals showing t-linear resistivity. *Science***339**, 804–807 (2013).23413351 10.1126/science.1227612

[13] Patel, A. A. & Sachdev, S. Theory of a Planckian metal. *Phys. Rev. Lett.***123**, 066601 (2019).31491164 10.1103/PhysRevLett.123.066601

[14] Franchini, C., Reticcioli, M., Setvin, M. & Diebold, U. Polarons in materials. *Nat. Rev. Mater.* **6**, 560–586 (2021).

[15] Murthy, C., Pandey, A., Esterlis, I. & Kivelson, S. A. A stability bound on the t-linear resistivity of conventional metals. *Proc. Natl Acad. Sci.***120**, e2216241120 (2023).36634139 10.1073/pnas.2216241120PMC9934301

[16] Allen, P. B. The electron-phonon coupling constant. *Tc***500**, 45 (2000).

[17] Mooij, J. Electrical conduction in concentrated disordered transition metal alloys. *Phys. Status Solidi (a)* **17**, 521–530 (1973).

[18] Chen, L. et al. Strong electron-phonon interaction in 2d vertical homovalent iii-v singularities. *ACS Nano***14**, 13127–13136 (2020).32960037 10.1021/acsnano.0c04702

[19] Schackert, M. et al. Local measurement of the Eliashberg function of pb islands: Enhancement of electron-phonon coupling by quantum well states. *Phys. Rev. Lett.***114**, 047002 (2015).25679904 10.1103/PhysRevLett.114.047002

[20] Lozano, D. P. et al. Experimental observation of electron-phonon coupling enhancement in sn nanowires caused by phonon confinement effects. *Phys. Rev. B* **99**, 064512 (2019).

[21] Zhang, H. et al. Origin of charge transfer and enhanced electron–phonon coupling in single unit-cell FeSe films on srtio3. *Nat. Commun.***8**, 214 (2017).28790304 10.1038/s41467-017-00281-5PMC5548863

[22] Staechelin, Y. U., Hoeing, D., Schulz, F. & Lange, H. Size-dependent electron-phonon coupling in monocrystalline gold nanoparticles. *ACS Photonics* **8**, 752–757 (2021).

[23] Pomarico, E. et al. Enhanced electron-phonon coupling in graphene with periodically distorted lattice. *Phys. Rev. B* **95**, 024304 (2017).

[24] Giri, A. et al. First-principles determination of the ultrahigh electrical and thermal conductivity in free-electron metals via pressure tuning the electron-phonon coupling factor. *Phys. Rev. B* **99**, 165139 (2019).

[25] Zhang, L. et al. Pressure-induced enhancement of electron-phonon coupling in superconducting Cac_6_ from first principles. *Phys. Rev. B* **74**, 184519 (2006).

[26] Lanzillo, N. A., Thomas, J. B., Watson, B., Washington, M. & Nayak, S. K. Pressure-enabled phonon engineering in metals. *Proc. Natl Acad. Sci.***111**, 8712–8716 (2014).24889627 10.1073/pnas.1406721111PMC4066520

[27] Ying, J. et al. Record high 36 k transition temperature to the superconducting state of elemental scandium at a pressure of 260 gpa. *Phys. Rev. Lett.***130**, 256002 (2023).37418707 10.1103/PhysRevLett.130.256002

[28] Wang, Z. et al. Tailoring the nature and strength of electron-phonon interactions in the SrTiO3 (001) 2d electron liquid. *Nat. Mater.***15**, 835–839 (2016).27064529 10.1038/nmat4623

[29] Arbouet, A. et al. Electron-phonon scattering in metal clusters. *Phys. Rev. Lett.***90**, 177401 (2003).12786103 10.1103/PhysRevLett.90.177401

[30] Yu, S., Zhang, J., Tang, Y. & Ouyang, M. Engineering acoustic phonons and electron-phonon coupling by the nanoscale interface. *Nano Lett.***15**, 6282–6288 (2015).26313532 10.1021/acs.nanolett.5b03227

[31] Maji, T. K. et al. Electrical resistance in a composite of ultra-small silver nanoparticles embedded in gold nanostructures: Implications for interface-enabled functionality. *ACS Appl. Electron. Mater.* **5**, 2893–2901 (2023).

[32] Kulik, I. On the determination of *α*^2^*f*(*ω*) in metals by measuring i-v characteristics of ‘wide’ (non-ballistic) point-contact junctions. *Phys. Lett. A* **106**, 187–190 (1984).

[33] Naidyuk, Y. G. & Yanson, I. K. *Point-contact spectroscopy*, vol. 145 (Springer Science & Business Media, 2005).

[34] Mott, D. M., Anh, D. T. N., Singh, P., Shankar, C. & Maenosono, S. Electronic transfer as a route to increase the chemical stability in gold and silver core-shell nanoparticles. *Adv. Colloid Interface Sci.***185–186**, 14–33 (2012).22999044 10.1016/j.cis.2012.08.007

[35] Mulvaney, P., Linnert, T. & Henglein, A. Surface chemistry of colloidal silver in aqueous solution: observations on chemisorption and reactivity. *J. Phys. Chem.* **95**, 7843–7846 (1991).

[36] Thi Ngoc Anh, D., Singh, P., Shankar, C., Mott, D. & Maenosono, S. Charge-transfer-induced suppression of galvanic replacement and synthesis of (Au@Ag)@Au double shell nanoparticles for highly uniform, robust and sensitive bioprobes. *Appl. Phys. Lett.* **99**, 073107 (2011).

[37] Yadav, V., Jeong, S., Ye, X. & Li, C. W. Surface-limited galvanic replacement reactions of pd, pt, and au onto ag core nanoparticles through redox potential tuning. *Chem. Mater.* **34**, 1897–1904 (2022).

[38] Mandal, S., Soundararajan, S., Jain, M. & Krishnamurthy, H. R. Possibilities for enhanced electron-phonon interactions and high-*T*_*c*_ superconductivity in engineered bimetallic nano-structured superlattices. arXiv 2408.15820 **[cond-mat.mes-hall]** (2024).

[39] Ziman, J. Principles *of the Theory of Solids* (Cambridge University Press, 1972). https://books.google.co.in/books?id=o4woMNO-C3sC.

[40] Suzuki, S. & Toyozawa, Y. Coexistence of itinerant electrons and self-trapped electrons. *J. Phys. Soc. Jpn.* **59**, 2841–2847 (1990).

[41] Jaime, M. et al. Coexistence of localized and itinerant carriers near t c in calcium-doped manganites. *Phys. Rev. B* **60**, 1028 (1999).

[42] Hao, X., Wang, Z., Schmid, M., Diebold, U. & Franchini, C. Coexistence of trapped and free excess electrons in SrTio3. *Phys. Rev. B* **91**, 085204 (2015).

[43] Ciuchi, S., Di Sante, D., Dobrosavljević, V. & Fratini, S. The origin of Mooij correlations in disordered metals. *npj Quantum Mater.* **3**, 44 (2018).

[44] Shepelyansky, D. L. Coherent propagation of two interacting particles in a random potential. *Phys. Rev. Lett.***73**, 2607–2610 (1994).10057103 10.1103/PhysRevLett.73.2607

[45] Andreev, A. V., Kivelson, S. A. & Spivak, B. Hydrodynamic description of transport in strongly correlated electron systems. *Phys. Rev. Lett.***106**, 256804 (2011).21770662 10.1103/PhysRevLett.106.256804

[46] Vool, U. et al. Imaging phonon-mediated hydrodynamic flow in wte2. *Nat. Phys.* **17**, 1216–1220 (2021).

[47] Saha, S. K. et al. Unconventional properties of engineered au-ag nanostructures. *Supercond. Sci. Technol.* **35**, 084001 (2022).

[48] Alemansour, H., Moheimani, S. O. R., Owen, J. H. G., Randall, J. N. & Fuchs, E. Ultrafast method for scanning tunneling spectroscopy. *J. Vac. Sci. Technol. B* **39**, 042802 (2021).

