# Supplementary material for: Engineering ultra-strong electron-phonon coupling and nonclassical electron transport in crystalline gold with nanoscale interfaces
Source: Nat Commun. 2025 Jan 2;16:61. doi: 10.1038/s41467-024-55435-z (PMC11696232; doi:10.1038/s41467-024-55435-z)
Supplement: Supplementary file 1 — Supplementary Information [file 41467_2024_55435_MOESM1_ESM.pdf]

# Supplementary Information
## Engineering ultra-strong electron-phonon coupling and nonclassical electron transport in crystalline gold with nanoscale interfaces

Shreya Kumbhakar[1,†,*], Tuhin Kumar Maji[1,†,*], Binita Tongbram[1], Shinjan Mandal[1], Shri Hari Soundararaj[1,3], Banashree Debnath[1], T. Phanindra Sai[1], Manish Jain[1], H.R. Krishnamurthy[1,4], Anshu Pandey[2], and Arindam Ghosh[1,*]

[1]*Department of Physics, Indian Institute of Science, Bangalore 560012, India*
[2]*Solid State and Structural Chemistry Unit, Indian Institute of Science, Bangalore 560012, India*
[3]*Materials Science and Engineering, University of California Riverside, Riverside, CA 92521, USA*
[4]*International Centre for Theoretical Sciences, Tata Institute of Fundamental Research, Bangalore 560012, India*

[†] ***authors contributed equally***
*and*
[*] ***Corresponding authors:***
*shreyak@iisc.ac.in*
*tuhinmaji@iisc.ac.in*
*arindam@iisc.ac.in*

## I. CHEMICAL SYNTHESIS

**Synthesis of AgNP and Ag@Au Nanohybrid:** AgNP was synthesized through the reduction of the silver precursor $AgNO_3$ with freshly produced frigid $NaBH_4$ as a reducing agent(See Ref. [1] for more detail). To control the size and uniformity of the AgNPs, Cetyltrimethylammonium Bromide (CTAB), a capping agent, was employed during the synthesis procedure. A solution mixture containing 10 ml of 0.1 M CTAB and 1 mL of 1 M $NH_4Br$ was prepared, with the pH marginally adjusted to be basic using 0.1 M aqueous NaOH. The reaction temperature was maintained at 40 °C throughout the synthesis. Subsequently, 80 µL of 0.1 M KI was added to the solution to achieve high-yield, monodispersed AgNPs of the desired size. The introduction of $AgNO_3$ (1 mM) into the reaction mixture caused a reduction in solution opacity, gradually turning it from clear to whitish turbid with time, owing to the formation of insoluble AgX (X= Halide) clusters. The 'wait time' played a crucial role in controlling the size and uniformity of the synthesized AgNPs. After a specific 'wait time', a 0.1 M solution of the reducing agent $NaBH_4$ was slowly added to the reaction mixture, resulting in an immediate color change from whitish to yellow, indicating the formation of AgNPs. The formation of AgNP has been validated further by the emergence of a characteristic surface plasmon resonance (SPR) peak of AgNPs at $\sim 393$ nm.

Subsequently, the gold precursor $HAuCl_4$ (1 mM) was promptly added to the reaction mixture, leading to a color transition from yellow to brown, indicative of Ag@Au NH formation. The UV-Vis spectra also display a red shift in the system's overall absorbance peak, indicating the production of Ag@Au NH. To terminate the reaction and induce precipitation of the nanohybrids, isopropyl alcohol (IPA) was added to the reaction mixture. The solution containing the synthesized nanohybrid was then subjected to centrifugation at approximately $10,000$ rpm for $15 - 20$ minutes, followed by the removal of the supernatant and collection of the solid sample for further processing.

**Synthesis of AuNP:** AuNPs were produced following a similar protocol to the AgNPs synthesis. In the present instance, we have used $HAuCl_4$ as the precursor of gold in place of Ag-precursor $AgNO_3$ keeping the other parameter fixed. In this case, 1 mM $HAuCl_4$ was added to a reaction mixture containing the same concentrations of CTAB, NaOH, $NH_4Br$, and KI as specified before. The reaction mixture was subsequently treated with a freshly made ice-cold $NaBH_4$ (0.1 M) solution. The colour of the solution changed to reddish-purple, indicating the formation of AuNPs. The solution containing the as-synthesized AuNP was then centrifuged at $10,000$ rpm for 15 minutes. The excess liquid was discarded, and the solid sample was extracted for further investigation.

## II. CHARACTERIZATION

### A. Optical Characterization

In order to monitor the process of Ag@Au-NH synthesis, we have monitored the reaction using an in-situ UV-Vis absorption measurement setup. Upon the formation of AgNP, a strong peak emerges at $\sim 393$ nm [2], corresponding to the localized surface plasmon resonance (LSPR) peak of ultrasmall AgNPs. As the size of the AgNPs in the solution increases, the LSPR experiences a gradual redshift over time. The introduction of $HAuCl_4$ leads to further redshifting of the LSPR peak as Au clusters begin to form (indicated by the yellow line). This redshift (See Fig. S1 below) continues, eventually resulting in the LSPR peak shifting towards gold peak ($\sim 524$ nm) [3] after a sufficiently long time. The observed long-term redshift in the LSPR can be attributed to the formation of a thicker shell and the gradual alloying of Ag and Au at the surface due to interdiffusion [4]. Notably, interdiffusion becomes more prominent as the shell thickness increases, particularly when the core material diameter is equal to or less than 4.5 nm [4]. To prevent excessive interdiffusion and maintain a well-defined AgNP surface, we employ a strategy of adding isopropyl alcohol (IPA) within 30 seconds of introducing $HAuCl_4$. This addition precipitates the nanohybrids, resulting in individual Ag@Au NH particle sizes of approximately $15 - 20$ nm. The corresponding LSPR peak, represented by the yellow trace exhibits minimal redshift compared to the pristine Ag LSPR. The relatively small redshift might be related to the formation of hybrid nanoparticles, in which the AgNPs are partly covered while retaining their structural integrity.

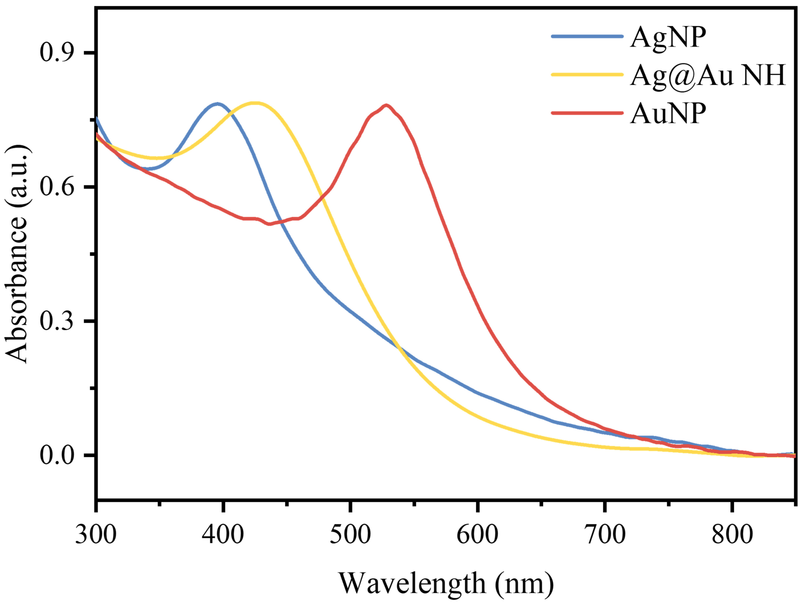


Fig. S1. **UV-Vis spectroscopy**: UV-Vis spectra of AgNPs (blue trace), Ag@Au nanohybrid at the point of reaction stopping (yellow trace), and AuNPs (red trace). The reaction was halted at a stage where the AgNP peaks dominate, preserving the surface integrity of the AgNPs.

## B. Structural Characterization

### *TEM of AgNP*

The characterization of the size distribution and morphology of AgNPs was performed using transmission electron microscopy (TEM). The TEM images revealed that the AgNPs exhibited a spherical shape, with well-defined boundaries and uniformity in their overall structure. To quantitatively analyze the size distribution of the AgNPs, measurements were taken from multiple TEM images. The average radius of the AgNPs was determined to be approximately $1.1 \pm 0.05$ nm (Fig. S2a), indicating a relatively small size range for the nanoparticles. This narrow size distribution was further confirmed by the observation of a Gaussian distribution in the particle size data (Fig. S2b), which signifies a statistically significant clustering around the average size. Figure S2c displays a representative image of the AgNP, providing additional confirmation of its spherical morphology and well-defined crystalline structure. This analysis provides evidence for the monodispersity and spherical nature of the AgNPs.

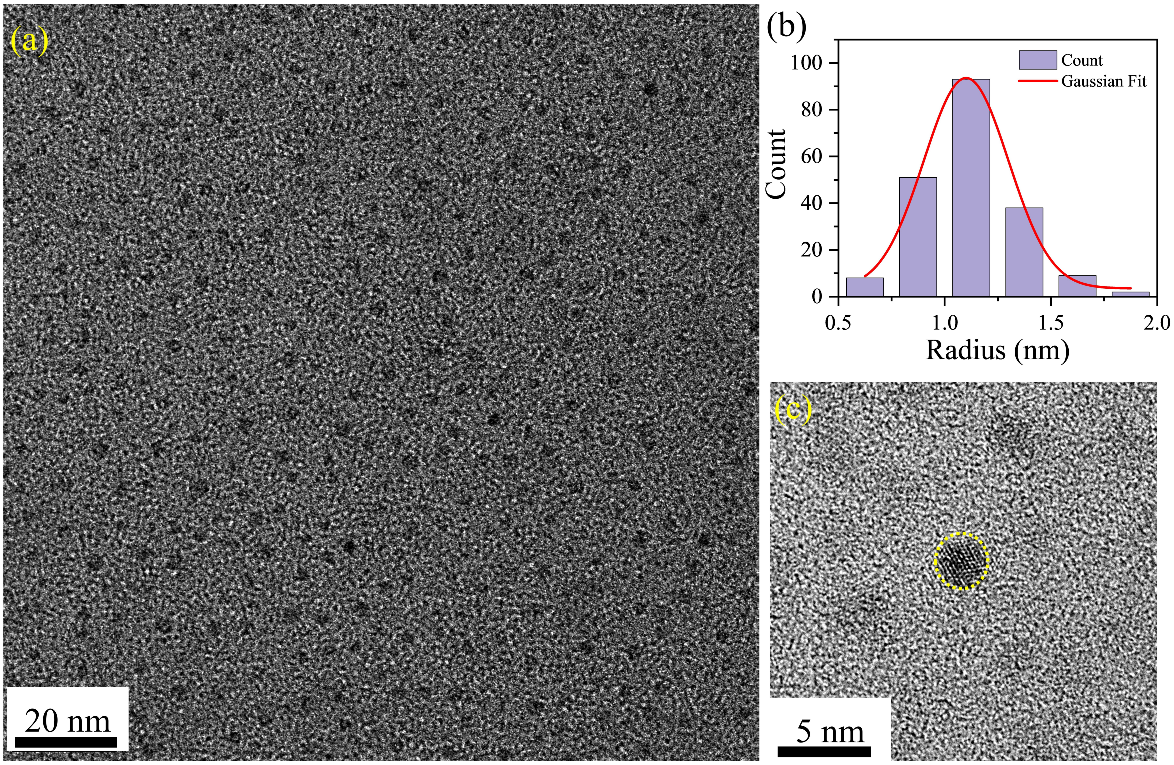


Fig. S2. **Size Distribution of AgNPs:** (a)TEM images of as-synthesized AgNPs, indicating the nearly monodisperse nature of the AgNPs. (b) The size distribution and Gaussian fitting of monodispersed AgNPs. The AgNP particle radius ($r_{\mathrm{Ag}}$) is approximately 1.1 nm. (c) A typical HRTEM image of a free-standing AgNP shows high crystalline and spherical nature.

***TEM of Ag@Au nanohybrid***

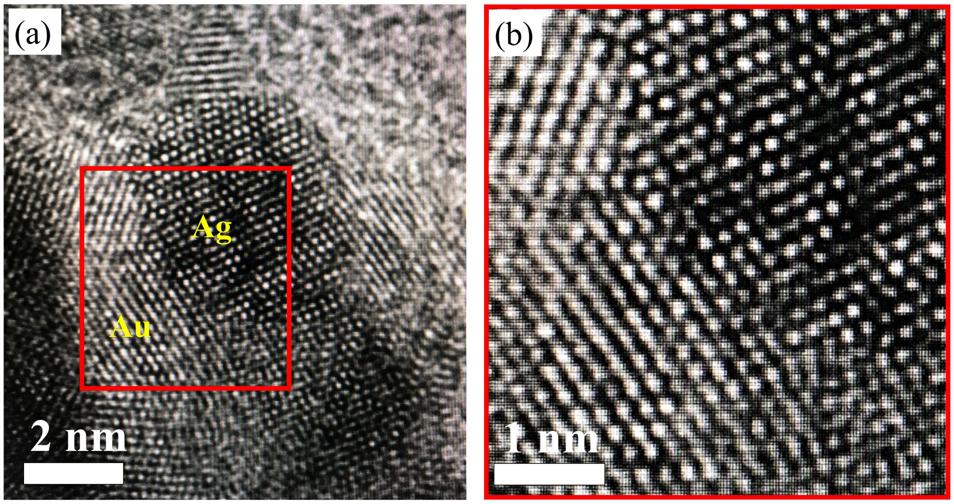


Fig. S3. **High-Resolution Transmission Electron Microscopy (HRTEM) Image of Ag@Au Interface:** (a) presents a high-resolution image of the Ag@Au nanohybrid with atomic precision, where the darker region represents the AgNPs surrounded by the lighter region, the Au matrix. The zoomed-in image (b) focuses on the interface between the AgNPs and the Au matrix, marked by a red squre in (a), revealing a well-defined sharp boundary between the AgNPs and the Au matrix. This indicates that the AgNPs are encapsulated by the Au matrix, forming a core-shell structure. The absence of visual line defects in these images indicates the high-quality crystalline nature of the nanohybrid.

### *X-Ray Diffraction (XRD)*

X-ray diffraction (XRD) analysis was performed using a Rigaku Miniflex-II instrument (Rigaku, Shibuya-ku, Japan) with Cu K$\alpha$ radiation, employing a step size of 0.02. The XRD patterns of the synthesized gold nanoparticles (AuNPs) exhibited distinctive peaks at $2\theta$ angles of $38.1°, 44.3°, 64.5°$, and $77.7°$. These peaks corresponded to the standard Bragg reflections $(111), (200), (220)$, and $(311)$, respectively[5], indicating the face-centered cubic (fcc) lattice structure of the gold nanocrystals. The intense diffraction observed at the $38.1°$ peak indicated the preferred growth orientation of the zero-valent gold in the (111) crystallographic direction. In the case of the Ag@Au nanohybrids, the XRD peaks observed were in alignment with the positions of the peaks observed in the pure AuNPs[6]. The preservation of the crystalline structure in the core-shell nanohybrids is indicated by the presence of the corresponding peaks at the same positions as the AuNPs.

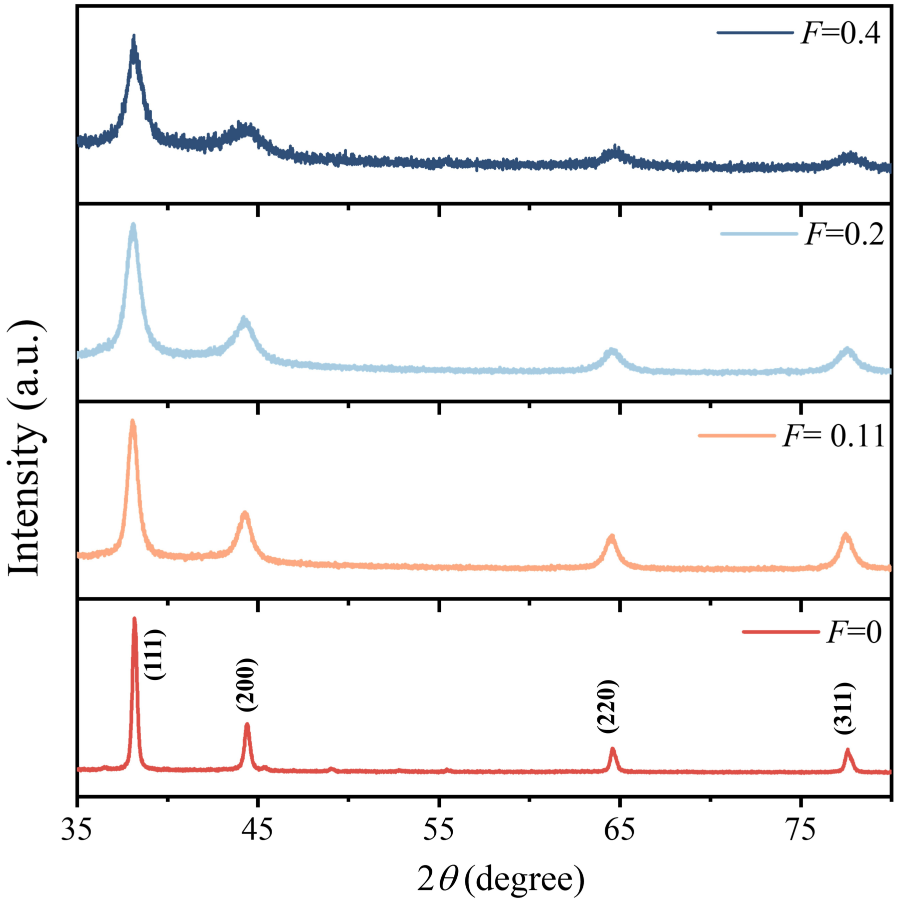


Fig. S4. **XRD of Ag@Au nanohybrids**: XRD pattern of AuNP ($F = 0$) and Ag@Au nanohybrid of different $F$ ratios, where $F = V_{\mathrm{Ag}}/(V_{\mathrm{Ag}} + V_{\mathrm{Au}})$. The XRD pattern of the hybrid structure shows no shift in peak positions compared to the pristine AuNPs, suggesting that the crystalline structure remains unaltered in the hybrid material.

### *X-ray photoelectron spectroscopy (XPS)*

The X-ray photoelectron spectroscopy (XPS) measurements have been performed to understand the electronic structure in the Ag@Au NH. The high-resolution scan of Au and Ag peaks shows doublet peaks from spin-orbit splitting. The high-resolution Au $4f$ spectrum (Extended Data Fig. 7) exhibits peaks at $\sim$ 87.9 and 84.1 eV, which can be attributed to Au $4f_{5/2}$ and Au $4f_{7/2}$, respectively [7]. These findings confirm the presence of metallic Au in the sample consistent with previous reports. On the other hand, Ag $3d$ scans for AgNPs shows a doublet of Ag $3d_{3/2}$ ($\sim$ 373.7 eV) and $3d_{5/2}$ ($\sim$ 367.7 eV). All the XPS peaks are normalized, with respect to the C=C peak at 284.5 eV. We observe a gradual decrease in the binding energy (BE) of the Au $4f$ core levels and an increase in that of the Ag $3d$ levels with increasing $F$.
To interpret the BE shift of the core electron energy levels we follow the electrostatic potential energy model given by, [8–10]

$$\Delta E_i = Kq_i + \frac{\Sigma q_j}{R_{ij}} \tag{S1}$$

where, $q_i$ is the net charge on atom $i$, $k$ is the Coulomb repulsion integral between core and valence states, and $R_{ij}$ is the distance between the nuclei $i$ and $j$. This model requires only the calculation of charges and implies that the chemical shifts of inner electrons are entirely determined by the valence electron distribution in the ground state.

A positive (negative) shift is predicted in cases where the net charge on the site of interest is positive (negative). Although the simplicity of the electrostatic potential model is appealing, its limitations are well documented [11, 12]. One illustrative example is the BE shift of binary alloys [13] where, for example, negative shifts have been measured [14] and calculated [13] for both Pd and Ag in random Pd-Ag alloys. The change in onsite electrostatic potential is one part of what generally is referred to as *initial-state effects*, which also include changes in the Fermi energy, intra-atomic charge transfer, and charge redistribution owing to bond rehybridization. In addition to initial-state effects, the photoemission experiment measures electronic relaxation around the created core hole. This relaxation ($\Delta E_i^r$) is often referred to as *final state effect.* A more accurate representation of the core level shift is consequently, given by [9]

$$E_i^{\mathrm{CLS}} = \Delta E_i - \Delta E_i^r \tag{S2}$$

While interpreting the chemical shift, one should thus carefully consider the effect of both the contributing terms, which are opposing in nature. For Ag, the final state effects are often significant [9]. In Au however, the BE shift for the $4f$ level is predominantly determined by the shift of electrostatic potential both in the bulk and nanostructured systems [15–18]. To understand the charge transfer in the Ag@Au films with the inclusion of Ag, we thus compare the BE shift of the Au $4f$ peaks with AuNP film as a reference level. Based on the electrostatic potential model, from the negative BE shift with increasing Ag, we conclude an effective decrease in the onsite potential in Au or in other words, electron doping in Au sites. For +1 oxidation, a BE shift of $1 - 1.5$ eV [7, 19–27] has been reported in many experiments. With DFT calculations possible oxidation states $\sim 0.1 - 0.2$ are reported in Au nanoclusters [28]. Based on the proportionality of the oxidation state($Ag^{-\delta}$) and the BE shift from the potential model Eq. [S1], we hence infer a charge transfer of $\delta \sim 0.3 - 0.4$ electron/Au atom for $F \sim 0.5$, where the BE shift is $\sim$ 0.4 eV. From charge neutrality, this naturally indicates hole doping in Ag. A dipolar structure at the Ag/Au interface also inhibits the reduction of Ag and hence can suppress galvanic replacement at Ag/Au interface,[29, 30] thus maintaining the integrity that we observe.

## III. FILM PREPARATION

The drop-cast method was utilized to fabricate the Ag@Au NH film on a glass substrate onto prepatterned Cr/Au contacts with a thickness of around 10 nm/60 nm. Before the drop-casting process, the solution containing Ag@Au NHs underwent centrifugation to remove any excess solvent. This step was crucial to ensure the removal of excess CTAB and getting pure solid material. The resulting solid residue was then subjected to vacuum drying at a pressure of approximately $\sim$ 1 mbar to eliminate any remaining solvent molecules. This drying process played a crucial role in enhancing the film's structural integrity and stability.

To prepare the film, the dried solid sample was first dispersed in chloroform and then subjected to sonication for 2 minutes. This step facilitated the formation of a well-dispersed solution of Ag@Au NHs in a chloroform medium, ensuring uniform deposition onto the pre-patterned contacts. The solution was then drop-cast onto the designated areas of the pre-patterned contacts, with proper care to control the quantity of the solution for each drop. After each drop, the sample was carefully dried at a temperature of 70 °C for 30 seconds. This thermal treatment enabled the

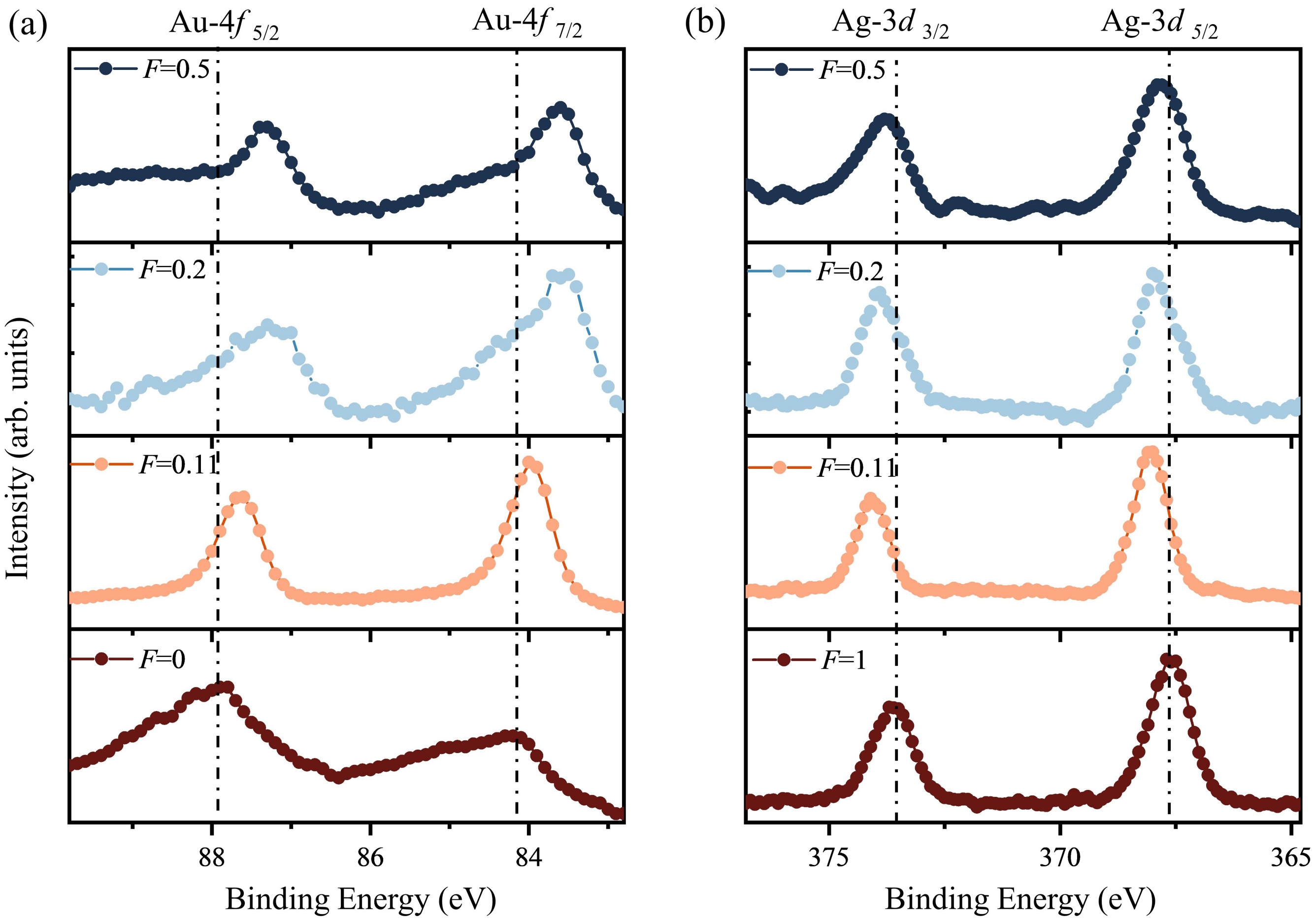


Fig. S5. **X-Ray Photoelectron Spectroscopy (XPS) spectrum of the Ag@Au nanohybrid:** High-resolution peak for (a) Au 4$f$, and (b) Ag 3$d$. The 4$f$ peaks of Au shift toward low binding energy, indicating $n$-type doping in the Au-matrix. At the same time, the 3$d$ peaks of Ag shift towards higher binding energy, which indicates that Ag is losing electrons to Au. All the peaks are normalized with respect to the C=C peak at 284.5 eV which is used as a charge correction reference.

removal of any residual solvent, resulting in enhanced adhesion and consolidation of the Ag@Au NHs on the substrate. After that, it was subjected to a thorough washing process. Firstly, the sample was rinsed with deionized (DI) water to remove any residual impurities and excess CTAB (Cetyltrimethylammonium Bromide), which was used as a capping agent during the synthesis process. This step ensured the removal of any unwanted substances and enhanced the cleanliness of the sample. Following the DI water rinse, the sample was washed with a 50 mM KOH solution. This solution served as a chemical agent to further eliminate any remaining CTAB and promote the formation of a chemically sintered cross-linked nanostructure. The interaction between the KOH solution and the sample's surface led to the formation of strong chemical bonds, resulting in a well-connected and compacted nanostructure. This procedure was critical to improve the structural integrity and properties of the synthesized material. To ensure the film's cleanliness and the removal of any remaining chemical residues, the sample was further cleaned with isopropyl alcohol (IPA) and dried again. This additional cleaning step enhanced the film's surface properties and eliminated any potential contaminants that could affect its performance. The aforementioned process was repeated ten times for each film, ensuring the formation of a well-controlled, uniform, and reproducible film structure. Optical profilometry was employed to measure the film thickness, yielding an average thickness ($t_f$) of approximately $3 \pm 0.5$ $\mu$m. This measurement provided valuable information about the film's dimensional characteristics.

The resulting film covered a circular area with a diameter of approximately 4 mm, effectively encapsulating the pre-patterned leads. This coverage ensured efficient electrical contact and facilitated subsequent measurements and characterization of the film's properties.

## IV. ELECTRICAL MEASUREMENT

### *Film Uniformity*

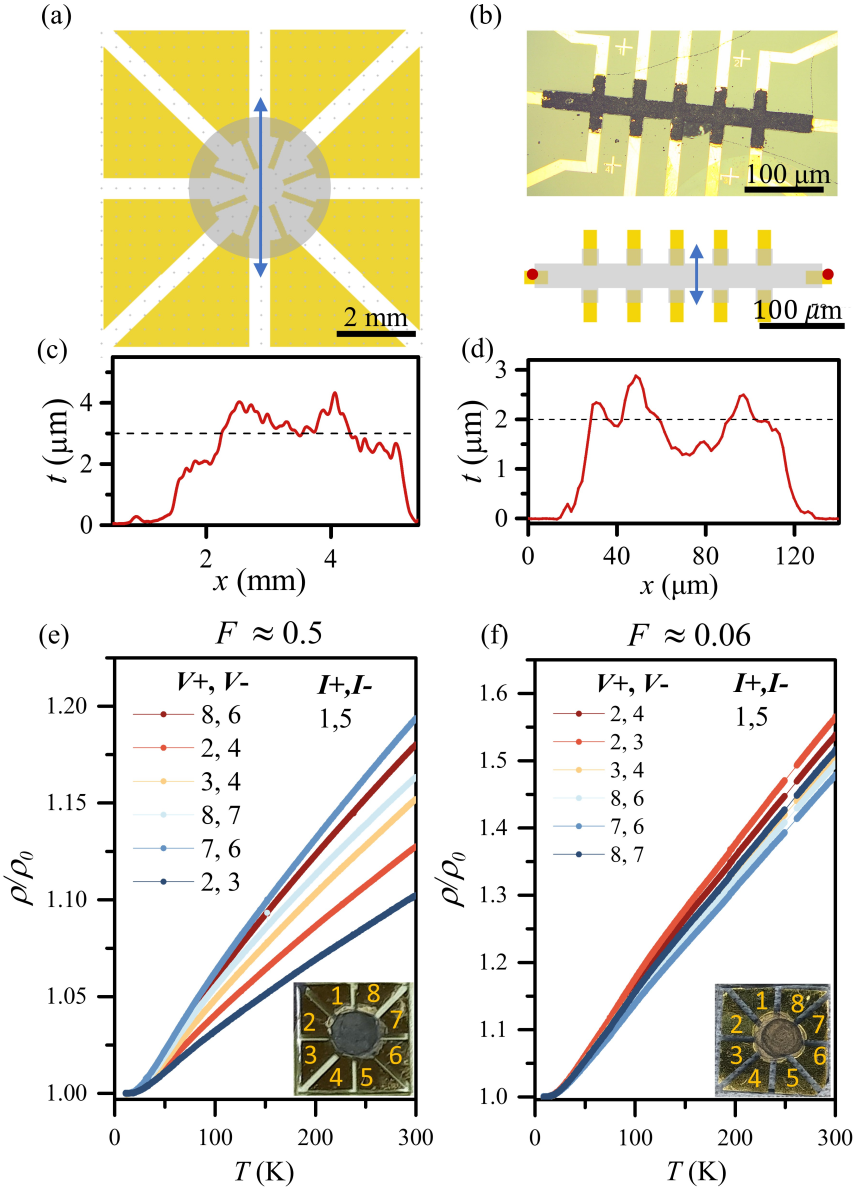


Fig. S6. **Film Uniformity:** (a). Schematic of Van der Pauw leads [31] used for most electrical measurements. The gold-coloured regions indicate the deposited Cr/Au electrodes and the grey circle indicates the dropcast film. (b) Optical image of a typical film on Hall bar leads. The bottom panel shows the schematic of the Hall bar leads, with red dots indicating the current contacts. The intermediate leads have been used for resistivity measurements as indicated by the green points of Fig. 1g of the main manuscript. All configurations of leads, provide identical results in resistivity measurements. (c) and (d) show the thickness profiles of typical films in Van der Pauw and Hall bar geometry measured via optical profilometry along the blue lines indicated in Fig. 1a and bottom panel of Fig. 1b, respectively. $x$ represents the spatial extent of the film. The average thickness is $2-3$ $\mu$m, represented by the dotted lines. (d) and (e) show the temperature ($T$) dependence of the resistivity ($\rho$) normalized by the residual resistivity ($\rho_0$, defined as the resistivity at $T \sim 6$ K) along multiple voltage channels for Ag filling $F = V_{\mathrm{Ag}}/(V_{\mathrm{Ag}} + V_{\mathrm{Au}}) = 0.5$ and 0.06, respectively. $I+, I-$, and $V+, V-$ indicate the current and voltage probes respectively. Insets show the typical images of the films at respective values of $F$. Variation of $\rho/\rho_0$ is within 10% along different channels which was observed in all films.

***Low-temperature data***

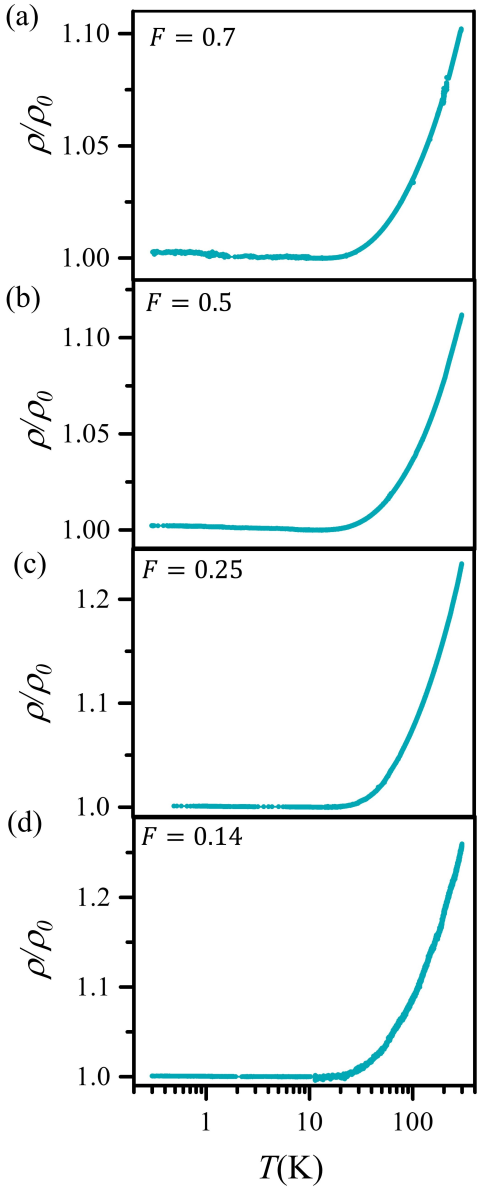


Fig. S7. **Low-temperature transport data:** (a), (b), (c), and (d) represent the temperature ($T$) dependence of the average normalized resistivity ($\rho/\rho_0$) for films with different Ag filling $F = 0.7, 0.5, 0.25$, and 0.14, respectively. The resistivity measurements were performed down to $T \sim 0.3$ K. Irrespective of $F$, no evidence of an upturn in resistance with decreasing $T$ (negative temperature coefficient of resistance), e.g., due to activation of electrons across inter-grain tunnel barrier through tunnelling or variable range hopping in case of weakly interconnected or granular assembly of nanoparticles [32–39], or any other disorder-mediated localization processes [40], was observed down to $T \sim 0.3$ K. The upper limit in the correction to conductivity ($\sigma$) for $F \sim 0.5$ is $\sim 100\ \Omega^{-1}.\text{m}^{-1}$, which is at least an order of magnitude smaller than that for bare Au film.

### *Residual resistivity with varying structural parameters*

.

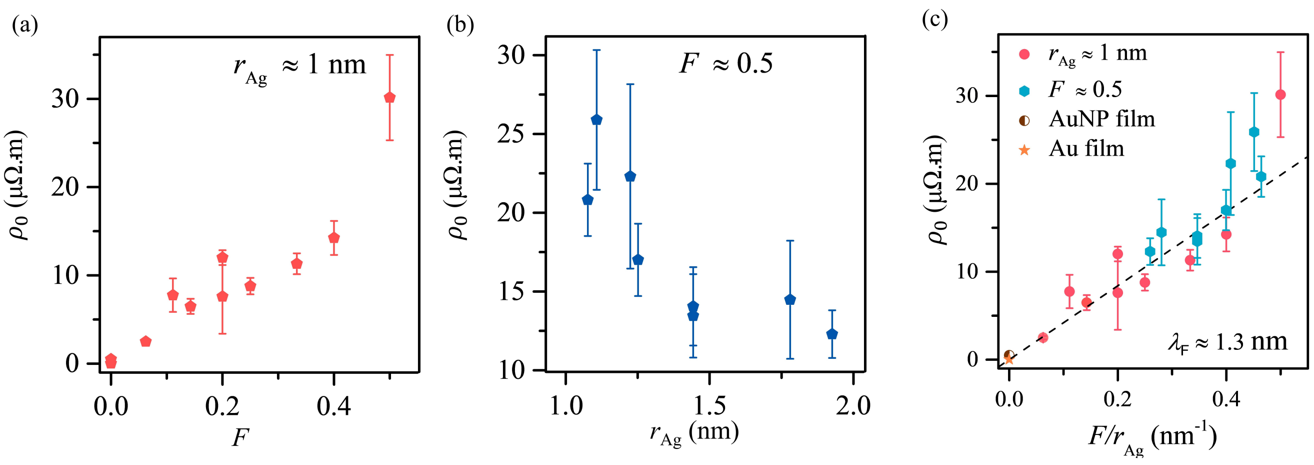


Fig. S8. **Variation of residual resistivity with the density of Ag-Au interface area at low fractional filling ($F \lesssim 0.5$):** (a) Residual resistivity ($\rho_0$), defined as the resistivity at $T \sim 6$ K, as a function of the volume fraction $F$ of Ag at the fixed AgNP radius ($r_{\mathrm{Ag}} \approx 1$ nm). (b) $\rho_0$ as a function of $r_{\mathrm{Ag}}$ at a fixed $F(\approx 0.5)$. (c) $\rho_0$ as a function of $F/r_{\mathrm{Ag}}$, the interface area of the AgNP per unit volume of the hybrid film. The collapse of all points onto a single linear behavior in $F/r_{\mathrm{Ag}}$ indicates that the Ag-Au interfaces are the dominant sources of scattering of electrons, as opposed to random atomic scale defects or alloying-related disorder. Within a simple Landauer-Büttiker formalism [1], where $\rho_0 \approx (h\lambda_{\mathrm{F}}^2/e^2) \times (F/r_{\mathrm{Ag}})$, we get the Fermi wavelength $\lambda_{\mathrm{F}} \sim 1.3$ nm (dashed line), which is within a factor of $\sim 2-3$ of that of crystalline Au. Error bars of resistivity shown in (a), (b) and (c) represent the standard deviation of the channel-to-channel statistics of resistivity in the same film.

### *Hall measurements*

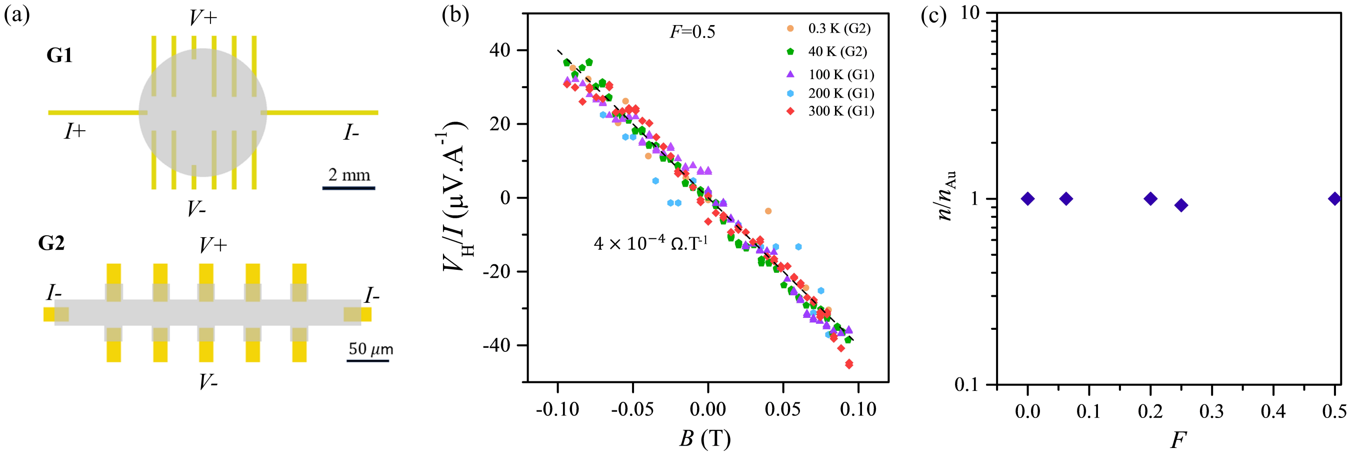


Fig. S9. **Hall Measurements:** (a) Top and bottom panels show the schematics of two different lead geometries (G1 and G2) used for Hall measurements. Yellow lines show the deposited Cr/Au electrodes and the light grey region is the dropcast film. $I+, I-$ and $V+, V-$ indicate the current and voltage probes respectively. (b) Low field hall measurements at different temperatures ($T$) varying from $0.3-300$ K illustrated with a typical film with Ag fraction $F = V_{\mathrm{Ag}}/(V_{\mathrm{Ag}} + V_{\mathrm{Au}}) = 0.5$. Hall resistance ($V_{\mathrm{H}}/I$, $V_{\mathrm{H}}$, and $I$ are the measured hall voltage and current, respectively) is plotted as a function of magnetic field ($B$) at different temperatures ($T$). The dotted line indicates the expected linear variation of the Hall resistance as a function of $B$, which was the case in all nanoparticle films measured during the course of this experiment. (c) The electron density $n$ ($\sim 10^{28}$ m$^{-3}$) for different $F$ normalized to the magnitude of that ($n_{\mathrm{Au}}$) obtained in bare AuNP film. This indicates that the electron density in the Ag@Au hybrids is the same to that of bare Au, irrespective of Ag fraction $F$.

## V. FITTING OF $\rho - T$ DATA

The resistivity ($\rho$) of metal with electron-phonon interaction playing the dominant role of scattering can be expressed in terms of the Bloch Grüneisen [41, 42] form as:

$$\rho(T) = \rho_0 + \rho_{\mathrm{BG}}(T) \tag{S3}$$

where $\rho_0$ is the residual resistivity, and

$$\rho_{\mathrm{BG}} = \frac{2\pi\lambda k_{\mathrm{B}}/\Theta_{\mathrm{D}}}{(n/m)e^2}\left(\frac{T}{\Theta_{\mathrm{D}}}\right)^5 \int_0^{\Theta_{\mathrm{D}}/T} \frac{x^5}{(e^x-1)(1-e^{-x})}dx \tag{S4}$$

is the Bloch Grüneisen form of resistivity arising from electron-phonon scattering. $\Theta_{\mathrm{D}}$, the Debye temperature, and $\lambda$, the electron-phonon coupling constant can be estimated by fitting the $\rho - T$ data with Eq. [S3]. As shown in Extended Data Fig. 5a, we have fitted the $\rho - T$ data of AuNP and AgNP films using Eq. [S3]. $\Theta_{\mathrm{D}} \sim 170$ K, and $\lambda \sim 0.45$ for Au, and $\Theta_{\mathrm{D}} \sim 190$ K and $\lambda \sim 1$ for Ag are estimated as fit parameters. The increased value of $\lambda$ as compared to the bulk value of $\sim 0.2$ for Au and Ag could be attributed to the nanostructuring in the film and increased electron scattering from the surfaces [43]. For Ag@Au films, Eq. [S3] cannot describe $\rho - T$ for the entire range of $T$. Extended data Fig. 5b shows that the transport data for $F = 0.5$ deviates from the low-temperature BG fit ($T \leq 100$ K) to the data. A two-component parallel channel [44–47] has been phenomenologically used to explain resistivity saturation in different materials. A non-classical conduction channel that is parallel to the channel of the 'ideal' metallic behavior of electron-phonon coupling is usually invoked to explain resistivity saturation in different materials. There are multiple theoretical models that lead to the resistivity of this new channel ($\rho_{\mathrm{sat}}$) in the order of the Mott-Ioffe-Regel resistivity [48] $\rho_{\mathrm{MIR}}$, although the microscopic origin and nature, for example, the temperature ($T$) dependence, vary dramatically across these models.

While a $T$-independent parallel channel has been indicated in both experiment and theory [44, 45], electron-phonon coupling through the Su–Schrieffer–Heeger model [47] predict an insulating-like parallel channel. Considering a conventional $T$-independent parallel channel with resistivity $\rho_{\mathrm{sat}}$, the net resistivity is represented as

$$\frac{1}{\rho} = \frac{1}{\rho_0 + \rho_{\mathrm{BG}}} + \frac{1}{\rho_{\mathrm{act}}} \tag{S5}$$

where, $\rho_{\mathrm{BG}}$ is the Bloch-Grüneisen form Eq. [S3] of resistivity arising from electron-phonon coupling, and $\rho_0$ is the residual resistivity. We find that this expression is not able to capture our data, as shown by the dotted line in Fig. [S20]. We show typical fits to the $\rho - T$ data with two possibilities.

### *Modified $T$-independent parallel channel*

As discussed in the main manuscript, a $T$-independent conduction channel that is parallel to the elastic part of scattering has been used to fit the data

$$\frac{1}{\rho - \rho_0} = \frac{1}{\rho_{\|}} + \frac{1}{\rho_{\mathrm{sat}}} \tag{S6}$$

We call this the modified $T$-dependent parallel channel. Details of the fit to $\rho - T$ data using this expression has been discussed in the Methods of main manuscript.

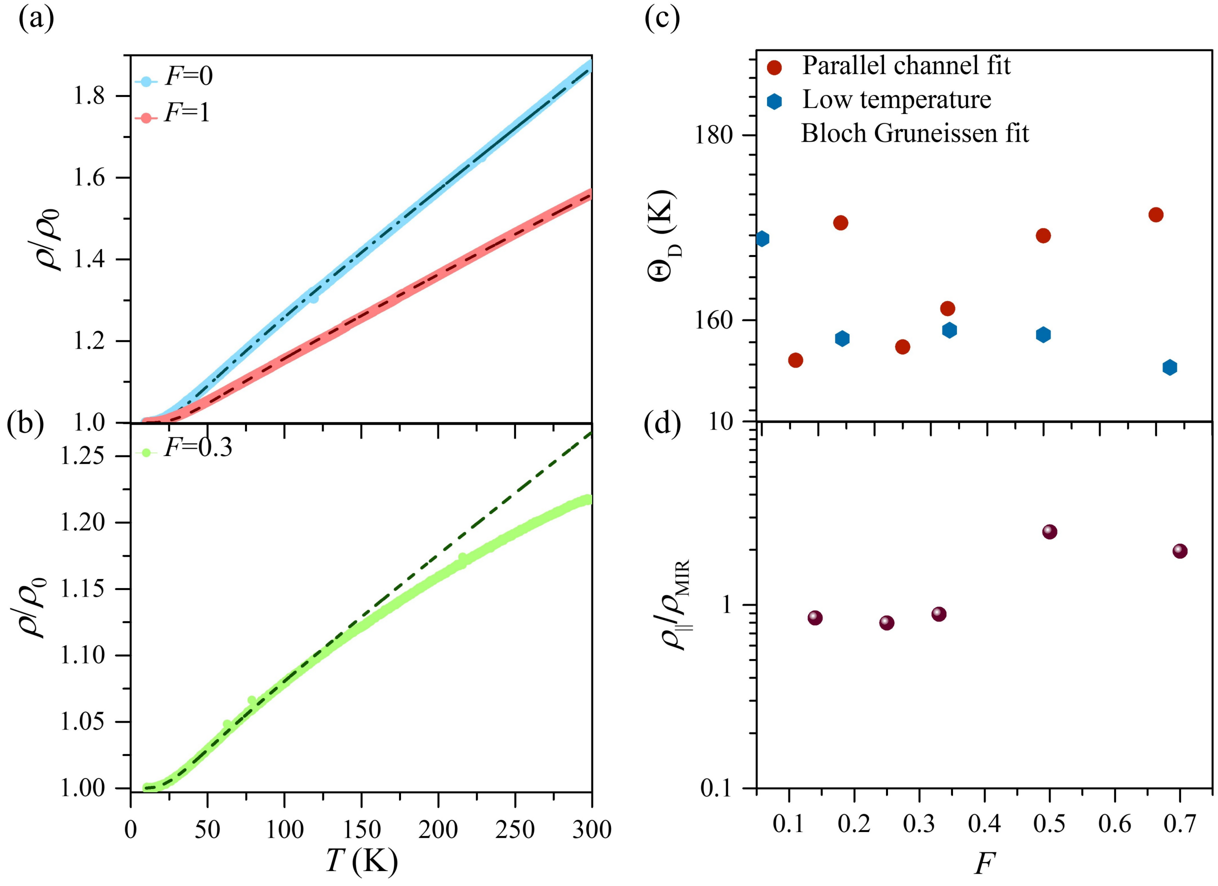


Fig. S10. **Fit of $\rho - T$ data:** (a) Bloch-Grüneisen fit (using Eq. [S3]) to $\rho - T$ data for Au nanoparticle ($F = 0$) and Ag nanoparticle ($F = 1$) films. Electron-phonon coupling constant $\lambda \approx 0.45$, and Debye temperature $\Theta_{\rm D} \approx 160$ K for Au, and $\lambda \approx 1$ and $\Theta_{\rm D} \approx 190$ K for Ag are derived as fit parameters. (b) Eq. [S3] is used to fit the $\rho - T$ data for $T \leq 100$ K in a film of Ag fraction $F = V_{\rm Ag}/(V_{\rm Ag} + V_{\rm Au}) = 0.33$ giving $\Theta_{\rm D} \sim 160$ K and $\lambda \sim 18$. However, extrapolation of the fit to high $T > 100$ K shows the deviation of the data from linearity. The sub-linearity of $\rho$–$T$ for $T \geq \Theta_{\rm D}$ naturally indicates the inadequacy of the Bloch-Grüneisen fit for films with $0 < F < 1$. (c) $\Theta_{\rm D}$ obtained from the Bloch-Grüneisen fit to $\rho - T$ data at $T < 100$ K and parallel channel model for entire $T$ range is plotted as a function of $F$. $\Theta_{\rm D}$ varies between $150 - 170$ K in all cases, which is close to that of Au ($\Theta_{\rm D,Au} \sim 180$ K). (d) $T$-independent parallel channel resistivity ($\rho_{||}$), obtained as a fit parameter in the parallel channel fit (Eq. [2] of main manuscript) to $\rho - T$ data is plotted with $F$ after normalizing with Mott-Ioffe-Regel resistivity [48] $\rho_{\rm MIR} \approx 3\pi^2\hbar^2 a/e^2 \approx 10$ $\mu\Omega$.m.

***Fitting with an thermally activated parallel channel***

We also consider an activated form of a parallel channel with resistivity $\rho_{\rm act}$, motivated by a theoretically predicted phonon-driven conduction channel [46, 47], which is insulating in nature.

$$\frac{1}{\rho} = \frac{1}{\rho_0 + \rho_{\rm BG}} + \frac{1}{\rho_{\rm act}} \tag{S7}$$

$$\rho_{\rm act} = \rho_{\rm A} e^{-\frac{E}{k_{\rm B}T}}$$

$\lambda, \Theta_{\rm D}, \rho_{\rm A}$, and $E$ are the electron-phonon coupling constant, Debye temperature, activated resistivity, and energy of activation respectively. These are derived as fitting parameters.

Fig. S4 shows $\rho/\rho_0$ ($\rho_0$, which is the residual resistivity is defined as the resistivity at $T \sim 6$ K) as a function of $T$ for a film with $F = 0.33$. The data is fitted by Eq. [S5] and Eq. [S7] as shown by the dotted, and solid lines, respectively. An activated form of the parallel channel Eq. ([S7]) fits the data better throughout the entire $T$-range than a conventional $T$-independent parallel channel given by, Eq. [S5]. The fit parameters derived are as follows: $\Theta_{\rm D} \approx 160$ K, $\lambda \approx 22$. $\rho_{\rm A} \approx 150 - 200$ $\mu\Omega$.m, and $E \approx 60$ meV.

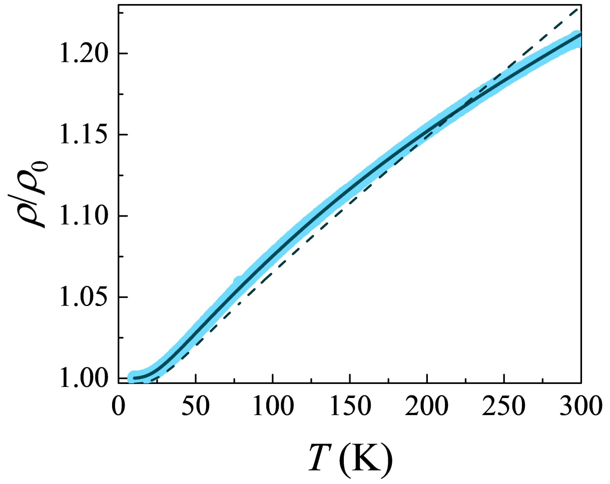


Fig. S11. **Fitting of $\rho - T$ data:** Average four-probe resistivity ($\rho$) of a film with $F = 0.33$, normalized with the residual resistivity $\rho_0$, defined as the resistivity at low temperature $T \sim 6$ K is measured as a function of temperature ($T$) from 300 K down to 6 K, as shown by the blue points. The dotted, and solid lines show fit to the data using Eq. [S5] and Eq. [S7], respectively.

## VI. SCHEMATIC OF THE CIRCUIT FOR POINT CONTACT SPECTROSCOPY MEASUREMENTS

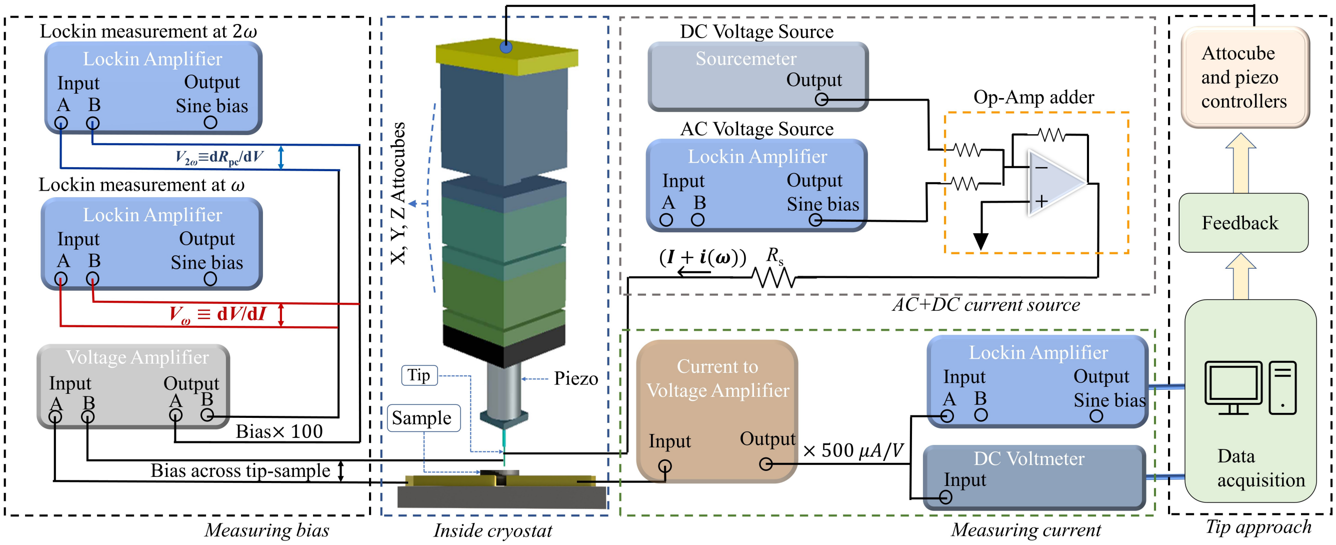


Fig. S12. **Schematic of the electrical circuit for point-contact measurements:** The tip is mounted on an attachment comprising of attocubes and piezo, which can control the movement of the tip in x,y and z direction down to the precision of a nanometer. The sample prepared on a pre-patterned glass substrate is loaded under the tip. This entire sample-tip chamber is loaded inside a home-built cryostat, which can go down to temperature $\sim 5$ K. Mixed AC+DC current ($I + i(\omega)$, $\omega$ being the frequency of the AC component) is passed to the tip by using a voltage source and series resistance $R_s$. The voltage across the tip-sample contact is then amplified with a voltage amplifier and measured with two lockin amplifiers simultaneously at frequencies $\omega$ and $2\omega$. The sample is grounded through a current-to-voltage amplifier, which converts even a small amount of current through the sample into a measurable voltage drop. The current through the sample is measured to monitor the tip-sample resistance.

## VII. POINT CONTACT SPECTROSCOPY OF THERMALLY DEPOSITED GOLD AND GOLD NANOPARTICLE FILMS

### A. Electron phonon coupling from point contact measurements

Point contact spectroscopy is the technique of extracting spectral information about the electron scattering mechanisms via transport through a narrow metallic constriction [49]. The narrowness of the constriction, quantified by its dimension $\sim d_{\rm PC}$, as compared to the mean free path of the system, $l_{\rm e}$ (elastic mean free path), and $l_{\rm in}$ (inelastic mean free path) determines the efficiency of the energy-resolving spectroscopy. Below, we provide a detailed derivation of the point contact spectrum.

#### *1. Ballistic regime*

When the dimension of the point contact (PC), $d_{\rm PC}$, is lesser than both the elastic and inelastic mean free paths *.i.e.* $d_{\rm PC} \ll l_{\rm el}, l_{\rm in}$, it is classified to be in the ballistic transport regime. Electrons passing through an ideal ballistic point-contact, biased with an external voltage $V$, gain excess energy $eV$ due to the absence of inelastic collisions at the junction. This energy is then dissipated by scattering, creating quasiparticle excitations. The scattering events result in the reflection of the electrons to the PC junction, resulting in a backflow current. This leads to nonlinear $I-V$ characteristics, whose higher order derivatives can be shown to correspond to the interaction function causing the scattering. In this case, the bias, $eV$, gives the spectral resolution of the interaction function. For metals, the electron-phonon interaction (EPI) is generally the dominant source of scattering. EPI function, also known as the Migdal Eliashberg function $\alpha^2\tilde{\mathcal{F}}(\omega)$ can thus be estimated from the non-linearities of $I-V$ characteristics in a point contact.

The point contact resistance $R_{\rm PC}$ [49] of has been derived by Wexler to be a combination of the ballistic Sharvin resistance ($R_{\rm sh} = 16\rho l/3\pi d_{\rm PC}^2$) and diffusive Maxwell resistance ($R_M = \rho/d$) as follows

$$\begin{aligned} R_{\rm PC} &= R_{\rm sh} + \Gamma\left(\frac{l_{\rm el}}{d_{\rm PC}}\right) R_{\rm M} \\ &= \frac{16\rho l}{3\pi d_{\rm PC}^2} + \Gamma\left(\frac{l_{\rm el}}{d_{\rm PC}}\right)\frac{\rho}{d} \end{aligned} \tag{S8}$$

where $\Gamma$ is a function of $l_{\rm el}/d$. When $l_{\rm el} \ll d_{\rm PC}$, which is the diffusive regime $\Gamma \approx 1$. $l$ in the above expression in the net mean free path of the system, which is often derived by Matheissen's rule in noble metals $l^{-1} = l_{\rm el}^{-1} + l_{\rm in}^{-1}$. We can estimate $l = v_F\tau$ ($\tau$ being the electron scattering time scale) from the Drude resistivity, $\rho = m/ne^2\tau = mv_F/ne^2 l$, which gives $R_{\rm sh} = 16mv_F/3\pi ne^2 d_{\rm PC}^2$. Hence, Eq. [S8] can be written as:

$$R_{\rm PC} = R_{\rm sh}(1 + \frac{3\pi d_{\rm PC}}{16 v_F \tau}) \tag{S9}$$

It is useful to note the ratio of $R_{\rm sh}$ to $R_{\rm M}$.

$$\frac{R_{\rm sh}}{R_{\rm M}} = \frac{16 l}{3\pi d_{\rm PC}} \tag{S10}$$

Differentiating Eq. [S9] gives

$$\frac{{\rm d}R_{\rm PC}}{{\rm d}V} = \frac{3\pi d_{\rm PC}}{16 v_F}\frac{\rm d}{{\rm d}V}(\frac{1}{\tau})R_{\rm sh} \tag{S11}$$

If the spectral dependence of the scattering comes entirely from the EPI, using Fermi-Golden rule we can write $\tau$ as

$$\begin{aligned} \frac{1}{\tau} &= \frac{2\pi}{\hbar}\int_0^{eV}\mathcal{G}(\epsilon)d\epsilon \\ \frac{{\rm d}(1/\tau)}{{\rm d}V} &= \frac{2\pi}{\hbar}e\mathcal{G}(\epsilon)|_{\epsilon=eV} \end{aligned} \tag{S12}$$

where $\mathcal{G}(\epsilon)$ represents the Migdal-Eliashberg spectral function. Substituting this in Eq. [S11] we have

$$\frac{1}{R_{\rm sh}}\frac{{\rm d}R_{\rm PC}}{{\rm d}V} \approx \frac{3ed_{\rm PC}}{\hbar v_F}\mathcal{G}(\epsilon)|_{\epsilon=eV} \tag{S13}$$

A more rigorous derivation by Kulik shows the PC spectrum to be

$$\frac{1}{R_0}\frac{\mathrm{d}R_{\mathrm{PC}}}{\mathrm{d}V} = \frac{8}{3}\frac{ed_{\mathrm{PC}}}{\hbar v_F}\mathcal{G}(\epsilon)|_{\epsilon=eV} \tag{S14}$$

$R_0$ is the point contact resistance at zero bias and is equal to the Sharvin contribution in the ballistic regime, $R_0 = R_{\mathrm{sh}}$. The electron-phonon coupling constant $\lambda$ is

$$\lambda = 2\int_0^\infty \frac{\mathcal{G}(\epsilon)}{\epsilon}d\epsilon \tag{S15}$$

However, it has been observed in experiments that the PC spectrum often shows a non-zero background at higher voltages, which is of the order of $\mathcal{G}(\epsilon)$. This is not captured by Eq. [S14] since $\mathcal{G}(\epsilon) = 0$ for $\epsilon \geq \hbar\omega_D$, $\omega_D$ being the Debye frequency. It has been shown by Gelder [50] for the first time and later demonstrated by Kulik [49, 51, 52] that if the point contact region is inhomogenous in geometry, inelastic collisions can happen at the narrowest part of the point contact. The relaxation of phonons spontaneously emitted in such collisions is a relatively slower process than the characteristic electron-phonon scattering time scales, leading to a phenomenon called *trapping/reabsorption of non-equilibrium phonons*. In other words, a non-equilibrium phonon gas at the PC orifice is generated due to the stimulated emission of phonons by the spontaneously emitted phonons. The electron scattering length scale by the non-equilibrium phonons, $l_r$ is smaller than characteristic electron-phonon scattering length scales $l_{\mathrm{e-ph}}$. The point contact spectrum [Eq. S14] in such cases is modified as [49]

$$\frac{1}{R_0}\frac{\mathrm{d}R_{\mathrm{PC}}}{\mathrm{d}V} = \frac{8}{3}\frac{ed_{\mathrm{PC}}}{\hbar v_F}[\mathcal{G}(\epsilon) + \gamma\int_0^\infty \frac{\mathcal{G}(\omega)}{\omega+\omega_0}d\omega + \frac{\gamma}{2}\frac{eV}{eV+\omega_0}\mathcal{G}(eV)] \tag{S16}$$

where $\gamma$ is a geometrical factor arising from the shape of the PC junction and is $\approx 0.58$ for a PC orifice. $\omega_0 = \omega_D l_r l_{\mathrm{e-ph}}/d_{\mathrm{PC}}^2$ is the phonon reabsorption frequency. The last two terms represent correction to the ballistic expression, Eq. [S14] due to a *background* signal.

### *2. Diffusive regime*

A point contact is described as diffusive if the dimension of the PC, $d_{\mathrm{PC}}$, is larger than the elastic mean free path ($l_{\mathrm{el}}$) but smaller than the inelastic relaxation length during the diffusion motion of electrons in the contact, $\Lambda = \sqrt{l_e l_{\mathrm{in}}/3}$, *.i.e.* $l_{\mathrm{el}} \ll d_{\mathrm{PC}} \ll \sqrt{l_e l_{\mathrm{in}}/3}$. This, of course, automatically implies $d < l_{\mathrm{in}}$. Kulik has shown that even in the diffusive regime, spectral information about the electron-phonon interaction can be obtained, and the proportionality between $\mathrm{d}R_{\mathrm{PC}}/\mathrm{d}V$ and $\mathcal{G}(\epsilon)$ is preserved. However, the intensity of the PC spectrum deviates from Eq. [S14] and is reduced by a factor $K$, known as the Knudsen factor, that depends on $l_{\mathrm{el}}/d$. For $l_{\mathrm{el}} \ll d$, it can be shown that $\langle K\rangle \sim (3\pi/4)l_{\mathrm{el}}/d_{\mathrm{PC}}$ in a PC orifice [49].

This modifies Eq. [S14] as:

$$\frac{1}{R_0}\frac{\mathrm{d}R_{\mathrm{PC}}}{\mathrm{d}V} = \frac{3\pi}{4}\frac{l_{\mathrm{el}}}{d_{\mathrm{PC}}}\frac{8ed_{\mathrm{PC}}}{\hbar v_F}[\mathcal{G}(\epsilon) + \gamma\int_0^\infty \frac{g(\omega)}{\omega+\omega_0}d\omega + \frac{\gamma}{2}\frac{eV}{eV+\omega_0}\mathcal{G}(eV)] \tag{S17}$$

For $l_e \ll d \ll l_{\mathrm{in}}$, $R_0 \approx R_M \approx \rho/d$. Also, $l \approx l_{\mathrm{el}}$Hence, we can write Eq. [S17] as follows:

$$\begin{aligned}\frac{1}{R_{\mathrm{sh}}}\frac{\mathrm{d}R_{\mathrm{PC}}}{\mathrm{d}V} &= \frac{R_M}{R_{\mathrm{sh}}}\frac{3\pi l_{\mathrm{el}}}{4d_{\mathrm{PC}}}\frac{8ed_{\mathrm{PC}}}{3\hbar v_F}[\mathcal{G}(\epsilon) + \gamma\int_0^\infty \frac{\mathcal{G}(\omega)}{\omega+\omega_0}d\omega + \frac{\gamma}{2}\frac{eV}{eV+\omega_0}\mathcal{G}(eV)] \\ &= \frac{3\pi d_{\mathrm{PC}}}{16 l_{\mathrm{el}}}\frac{3\pi l_{\mathrm{el}}}{4d_{\mathrm{PC}}}\frac{8ed_{\mathrm{PC}}}{3\hbar v_F}[\mathcal{G}(\epsilon) + \gamma\int_0^\infty \frac{g(\omega)}{\omega+\omega_0}d\omega + \frac{\gamma}{2}\frac{eV}{eV+\omega_0}\mathcal{G}(eV)] \\ &= \frac{3\pi^2}{8}\frac{ed_{\mathrm{PC}}}{\hbar v_F}[\mathcal{G}(\epsilon) + \gamma\int_0^\infty \frac{\mathcal{G}(\omega)}{\omega+\omega_0}d\omega + \frac{\gamma}{2}\frac{eV}{eV+\omega_0}\mathcal{G}(eV)]\end{aligned} \tag{S18}$$

Interestingly, this is equivalent to Eq. [S16] derived before, representing that $1/R_{\mathrm{sh}}\mathrm{d}R_{\mathrm{PC}}/\mathrm{d}V$, not $1/R_0\mathrm{d}R_{\mathrm{PC}}/\mathrm{d}V$, is the correct measure of $\tilde{g}(\epsilon)$. From Eq. [S16] and Eq. [S18], we observe that in both the ballistic and diffusive regime, the point contact spectrum $\mathrm{d}R_{\mathrm{PC}}/\mathrm{d}V$ leads us to the EPI function.
However, for strong reabsorption of phonons, $\omega_0 \to 0$ since as $d \gg l_{\mathrm{e-ph}}, l_r$. In such cases, the point contact spectrum

in both ballistic and diffusive regimes can be summarized in terms of the following expressions

$$\frac{1}{R_{\rm sh}}\frac{{\rm d}R_{\rm PC}}{dV} \approx \frac{3\pi^2}{8}\frac{ed_{\rm PC}}{\hbar v_F}\mathcal{G}(eV), \qquad \epsilon \leq \hbar\omega_D,\text{ weak phonon reabsorption} \tag{S19a}$$

$$\approx \frac{3\pi^2}{8}\frac{ed_{\rm PC}}{\hbar v_F}[\mathcal{G}(eV)[1+\frac{\gamma}{2}]+\gamma\frac{\lambda}{2}], \qquad \epsilon \leq \hbar\omega_D,\text{ strong phonon reabsorption} \tag{S19b}$$

$$\approx 0, \qquad \epsilon \geq \hbar\omega_D,\text{ weak phonon reabsorption} \tag{S19c}$$

$$\approx \frac{3\pi^2}{8}\frac{ed_{\rm PC}}{\hbar v_F}\frac{\gamma}{2}\lambda, \qquad \epsilon \geq \hbar\omega_D,\text{ strong phonon reabsorption} \tag{S19d}$$

Since $\gamma \sim 0.58$ for a circular orifice, the background signal at large bias directly corresponds to the electron-phonon coupling constant as:

$$\boxed{\frac{1}{R_{\rm sh}}\frac{{\rm d}R_{\rm PC}}{dV}(eV \gg \hbar\omega_D) \approx 1.1\frac{ed_{\rm PC}}{\hbar v_F}\lambda} \tag{S20}$$

### 3. *Thermal regime*

The thermal regime of point contact is defined as $d_{\rm PC} \ll l_{\rm el}, l_{\rm in}$. The electrons dissipate the energy at the constriction itself due to inelastic collisions, leading to the heating of the point contact. The temperature ($T$)-dependence of the resistivity $\rho$ is the origin of non-linearities in $I-V$ in such cases and can be represented as [49]:

$$I(V) = Vd\int_0^1 \frac{dx}{\rho(T\sqrt{(1-x^2)})|_{T=ev/3.63k_B}} \tag{S21}$$

This allows both calculation of the $I-V$ characteristic using $\rho(T)$ and vice-versa reconstruction of the $\rho(T)$ dependence in the constriction from the measured $I-V$ curve.

However, if the phonon contribution to the resistivity is less than the elastic scattering and the heat influx and heat outflux are both determined by electron-phonon collisions; then, a spatially dependent phonon distribution, related to $\mathcal{G}(\omega)$ will be introduced in the point-contact region, which is determined by the local temperature $T(r)$. This indicates that the argument of $\rho$ in the above equation involves a spatial integration over $T(r)$. Hence, the second-order derivative of $I-V$, ${\rm d}^2I/{\rm d}V^2 \approx 1/R_0^2{\rm d}R_{\rm PC}/{\rm d}V$ can be shown to be related to $T(r)$, and equivalently $g(\omega)$. The PC spectrum in such cases was derived [53] as follows:

$$\frac{1}{R_0}\frac{{\rm d}R_{\rm PC}}{{\rm d}V} = \frac{\pi\sqrt{3}m}{ne\hbar\rho}\int_0^\infty \frac{d\omega}{\omega}\mathcal{G}(\omega)S(eV/\omega) \tag{S22}$$

where,

$$S(x) = \frac{2\pi}{3}\frac{d^2}{dx^2}\int_0^{\pi/2}\frac{dy}{\sinh^2(\pi/\sqrt{3}x{\rm sin}y)}$$

It is interesting to note the difference between the two extreme regimes: ballistic and thermal. In the former case, ${\rm d}R_{\rm PC}/{\rm d}V$ is proportional to $\mathcal{G}(\epsilon)$, whereas in the latter, it is proportional to $\mathcal{G}(\epsilon)$ convoluted by a function of $S(\epsilon)$, which arises from non-equilibrium or thermal effects. Thus, the spectral information of the EPI is smeared out in the thermal regime.

For $eV \gg \hbar\omega_{\rm D}$, $S(eV/\hbar\omega) \approx 1$, at all finite values $\mathcal{G}(\omega)$. Hence, from Eq. [S22] we can write:

$$\begin{aligned}\frac{1}{R_0}\frac{{\rm d}R_{\rm PC}}{{\rm d}V}(eV \gg \hbar\omega_D) &= \frac{\pi\sqrt{3}m}{ne\hbar\rho}\int_0^{\hbar\omega_D}\frac{g(\omega)}{\omega}d\omega \\ &= \frac{\pi\sqrt{3}m}{2ne\hbar\rho}\lambda\end{aligned} \tag{S23}$$

Since $R_{\rm PC} \approx R_{\rm M} = \rho/d$ in the thermal regime, the above equation can be written as

$$\frac{1}{R_{\rm sh}}\frac{{\rm d}R_{\rm PC}}{{\rm d}V}(eV \gg \hbar\omega_D) = \frac{R_{\rm M}}{R_{\rm sh}}\frac{\pi\sqrt{3}m}{2ne\hbar\rho}\lambda = \frac{3\pi d_{\rm PC}}{16l}\frac{\pi\sqrt{3}m}{2ne\hbar\rho}\lambda = \frac{3\pi^2}{32}\frac{d_{\rm PC}m}{ne\hbar}\frac{ne^2}{mv_F}\lambda \tag{S24}$$

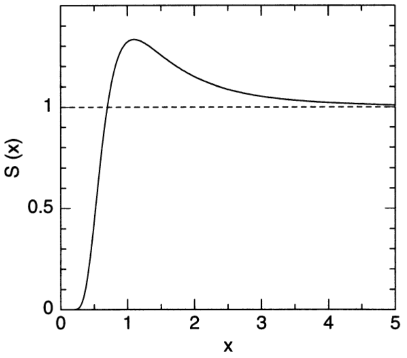


Fig. S13. Variation of $S(x)$

So we arrive at the expression:

$$\boxed{\frac{1}{R_{\mathrm{sh}}}\frac{\mathrm{d}R_{\mathrm{PC}}}{\mathrm{d}V}(eV \gg \hbar\omega_D) = 1.8\frac{ed_{\mathrm{PC}}}{\hbar v_F} \times \lambda} \tag{S25}$$

**Summary**

- In the ballistic and diffusive regimes, due to the absence of inelastic collisions at the point-contact the excess energy $eV$ gained by the electrons while passing the PC provides the spectroscopic probe to the EPI, as represented by Eq. 11a.
- In the presence of inhomogeneities near the point contact, the spectrum deviates from the equilibrium case discussed above in the ballistic and diffusive regimes. The non-equilibrium processes change the effective PC temperature and result in a non-zero background as well as lead to smearing of the spectral dependence, as can be observed from Eq. [S16], and Eq. [S18]. In fact, for significant phonon trapping/reabsorption ($\omega_0 \to 0$), the background signal ($\mathrm{d}R_{\mathrm{PC}}/\mathrm{d}V(eV \gg \hbar\omega_D)$) directly manifests the cumulative EPI, represented by $\lambda$ (Eq. [S20]).
- The ballistic and diffusive regimes are equilibrium conditions, where the non-equilibrium processes are considered as perturbations. Eq. [S19(b),(d)] is the extreme limit of the perturbation. The thermal regime is considered to be intrinsically in the non-equilibrium regime when the point contact is smaller in size compared to both the elastic and inelastic collisions. In such a case, the spectral information is entirely smeared. However, the background value similarly corresponds to the net EPC value, Eq. [S25].
- Eq. [S20,S25] can be summarized as

$$\frac{1}{R_{\mathrm{sh}}}\frac{\mathrm{d}R_{\mathrm{PC}}}{\mathrm{d}V}(eV \gg \hbar\omega_D) = f\frac{ed_{\mathrm{PC}}}{\hbar v_F} \times \lambda \tag{S26}$$

where the constant $f \sim 1.1-1.8$. A remarkable implication of this expression is the fact that in the presence of a non-equilibrium phonon distribution at the point contact, the background signal in the PC spectrum represents the $\lambda$, irrespective of the origin of this non-equilibrium gas, an inhomogenous PC (ballistic/diffusive) or a 'wide' PC (thermal). Physically, this means the scattering of electrons causing the non-linearities in $I-V$ at $eV \gg \hbar\omega_D$, is a cumulative contribution from the non-equilibrium phonons available at all energies below $\hbar\omega_D$. Hence, in all three regimes of PC transport, $\lambda$ can be extracted from the background signal of the PC spectrum given phonons are the dominant inelastic scatterers in the system.
Eq. [S26] can be rewritten as

$$d_{\mathrm{PC}}\frac{\mathrm{d}R_{\mathrm{PC}}}{\mathrm{d}V}|_{V\to\infty} = \frac{16f}{3\pi}\frac{m}{ne\hbar}\lambda \tag{S27}$$

The right side of the above equation is the PC spectrum normalized by the PC dimension and depends on physical entities intrinsic to the system. Eq. S27 can be inverted to derive the $\lambda$ as shown in Eq. [1] in the main manuscript.

$$\lambda = \frac{3\pi}{16f}\frac{ne\hbar}{m}\left[d_{\mathrm{PC}}\frac{\mathrm{d}R_{\mathrm{PC}}}{\mathrm{d}V}\right]_{V\to\infty} \tag{S28}$$

## B. Analysis of experimental results

### 1. *Estimation of the point contact diameter*

The typical residual resistivities of the film that we have measured vary within $\rho \sim 10^{-6} - 10^{-5}$ Ω.m. Assuming the fermi velocity of Au/Ag $\sim v_F \sim 1.4 \times 10^6$ m.s$^{-1}$, we get the mean free path to be $l = \sqrt{D\tau} \sim v_F\tau \sim 10^{-9} - 10^{-10}$ m, where $D = v_F^2\tau$ is the electron diffusivity and $\tau$, the scattering time is estimated from the Drude expression of resistivity, $\rho = m/ne^2\tau$. This is larger than the typical point contact (PC) diameter achieved experimentally by mechanical methods. Hence, we expect the resistance to primarily dominate the Maxwell contribution *.i.e.*, $R_{\mathrm{PC}} \sim \rho/d_{\mathrm{PC}}$. To establish this experimentally, we have performed the following experiment. The point contact resistance, $R_{\mathrm{PC}}$, was recorded in a film with an Ag-filling fraction, $F = 0.7$, while approaching the tip towards the sample with a z-piezo positioner. $R_{PC}$ kept on decreasing as the tip gradually approached the sample. Without loss of generality, the PC region can be modelled as a sphere of radius $d_{\mathrm{PC}}$, with the centre of the sphere located at the centre of the PC-plane. This is depicted schematically in Fig. S14(a), where the side view shows a conical tip touching the sample. As the tip is approached further with the piezo, the sphere effectively becomes larger, thus decreasing $R_{\mathrm{PC}}$. We have used a z-piezo from PI ceramics, whose axial displacement for a voltage increment of 0.1 $V$ is $\sim 0.6$ Å. Fig. b shows the $R_{\mathrm{PC}}$ as a function of the net piezo displacement from initial conditions, which is equivalent to the change in PC dia, $\Delta d_{\mathrm{PC}}$. The dotted line represents the Maxwell contribution, where $d_0$ is the starting PC diameter estimated as $d_0 \sim \rho/R_{\Delta d=0}$. The agreement of the dotted line with the experimental values represents the PC diameter to be accurately captured by the Maxwell resistance.

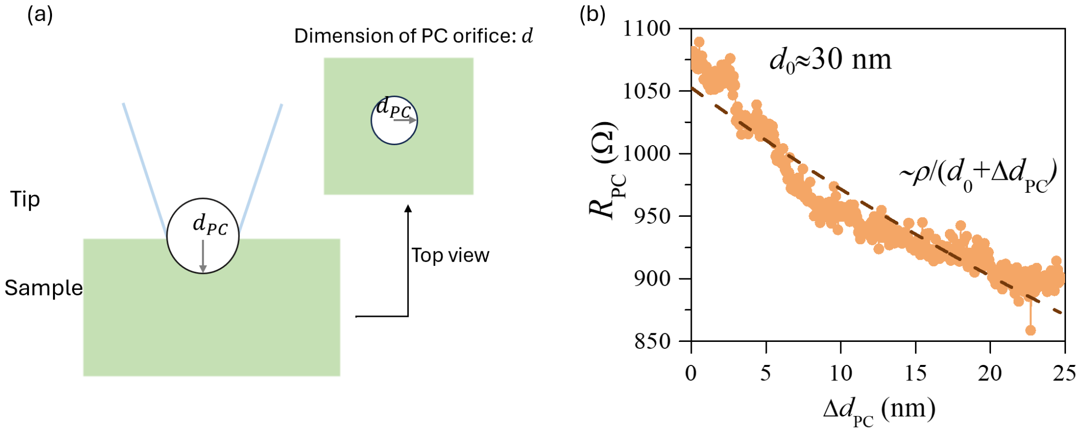


Fig. S14. **Estimation of the point-contact diameter:** (a) Schematic of the experimental realization of the tip-sample contact. (b) Point-contact (PC) resistance, $R_{\mathrm{PC}}$ as a function of the change in effective PC diameter, $\Delta d_{\mathrm{PC}}$.

### 2. *Estimation of the inelastic scattering length*

We have performed the point contact measurements at $T \sim 6$ K, where electron-phonon scattering is significantly suppressed since the Debye temperature in these films is $\sim 170$ K [shown in Extended Data Fig. 6 of main manuscript]. This is established by the saturation of $\rho(T)$ at these temperatures.[See Extended Data Fig. 3 of main manuscript]. To have a quantitative estimation of $l_{\mathrm{in}} \sim l_{\mathrm{e-ph}}$, let us look at the electron-phonon relaxation length that can be represented as: $l_{\mathrm{e-ph}} = v_F/\tau_{e-ph}$, where $\tau_{e-ph}^{-1} = (2\pi k_B/\hbar)\lambda T$. For $\lambda$ ranging from $0.2 - 20$ that is obtained by fitting the $\rho - T$ data, this varies from $10^{-7} - 10^{-9}$ nm, shown by red-colored points in Fig. S15(b). However, this is an overestimation of $l_{\mathrm{in}}$ since the scattering rate varies superlinearly, $\rho \sim T^5$ at lower temperatures, as can be understood from the asymptotic limit of the Bloch-Gruneissen expression of resistivity from electron-phonon scattering (discussed in Methods as well Supplementary Information). We have performed quantum transport measurements

at $T \sim 7$ K to extract $l_{\mathrm{in}}$. Fig. S15(a) shows the variation of the transverse resistance of a typical film with $F \approx 0.12$ in perpendicular magnetic fields ($B$) within $B \pm 5$ T. By fitting the data with the three-dimensional analogues of the Hikami-Larkin-Nagaoka expressions of weak localization/anti-localization [54], we extract the phase coherence length, $l_\phi$. This is equivalent to $l_{\mathrm{in}}$ under the assumption that there are no magnetic impurities in the system. The estimated $l_\phi$ is shown by blue-coloured points in Fig. S15(b). Further details on the quantum transport measurements will be shown in another manuscript (*Kumbhakar et.al*). We wish to highlight that $l_{\mathrm{in}}$ is the lower limit of $l_{e-ph}$ since we

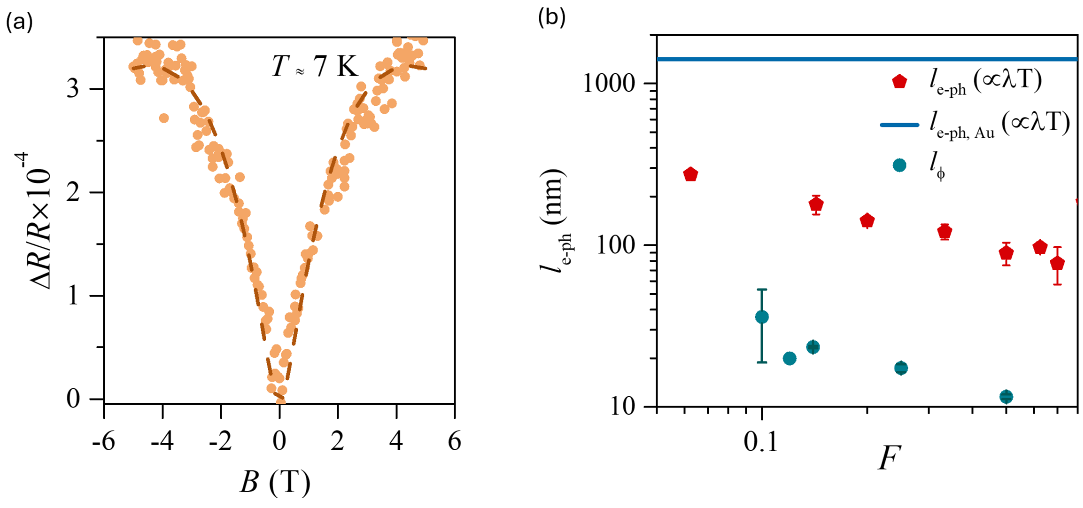


Fig. S15. **Inelastic scattering length:** (a) Magnetotransport in a film with $F = 0.12$ within perpendicular magnetic fields of $B \pm 5$ T. (b) Comparison of the inelastic scattering length ($l_{\rm in}$) computed in two different ways. Error bars in the red-coloured points have been computed from the error in resistivity, $\rho$, as shown in Fig. 1g of the main manuscript by error propagation. Error bars in the blue-coloured points represent the standard deviation of the channel-to-channel statistics of $l_\phi$, estimated from the fits to the quantum transport data of the particular channel.

did not eliminate the contribution of the inelastic processes from electron-electron scattering. This would mean that the point contact lies closer to the diffusive regime than indicated in Fig. S16. Hence, we have considered the value of $f \sim 1.1$ in the estimation of $\lambda$.

### *3. Transport regime of point contact*

Fig. S16 shows the variation of $l_{\rm el}, l_{\rm in}, \Lambda$, and $d_{\rm PC}$ with Ag-filling fraction $F$ at $T \sim 6-8$ K. $d_{\rm PC}$ shown in the figure corresponds to the dimension of the point contact orifice for the spectrum, shown in Fig. 2c of the main manuscript. $l_{\rm in}$ has been derived from the quantum transport measurements as described above. We observe $l_{\rm el}, \Lambda \ll d_{\rm PC} \leq l_{\rm in}$, indicating that the point contact is at the crossover between the diffusive and the thermal regimes., which we call the *quasi-thermal* regime. For estimating $\lambda$ from Eq. [S28], we have used $f \sim 1.1$, which is the limit for diffusive transport.

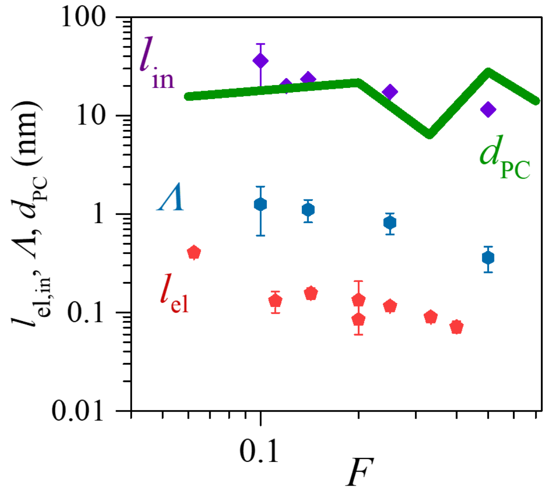


Fig. S16. Comparison of elastic ($l_{\rm el}$), inelastic ($l_{\rm in}$), electron diffusion ($\Lambda = \sqrt{l_{\rm el} l_{\rm in}/3}$) length scales, and the point contact diameter ($d_{\rm PC}$) for different values of Ag-filling $F$ at temperature $T \sim 6-8$ K. For $F \gtrsim 0.3, l_{\rm el}$ becomes lesser than the interatomic spacing, indicating the inapplicability of the Drude expression at these resistivities to estimate the elastic scattering length. Error bars in $l_{\rm el}$ have been computed from the error in resistivity, $\rho$, as shown in Fig. 1g of the main manuscript by error propagation. $l_{\rm in}$ shown here corresponds to the phase breaking length, $l_\phi$ estimated from quantum transport measurements, shown in Fig. S15. Error bars in $\Lambda$ have been computed from the errors in $l_{\rm el}$, and $l_{\rm in}$ by error propagation.

### 4. *Consistency of the analysis*

We present here the normalized point contact spectrum, $\rho/R_{\rm PC}\ {\rm d}R_{\rm PC}/{\rm d}V$ measured at different values of zero bias $R_{\rm PC,0}$ at different positions at temperatures $T \sim 6-8$ K. The dotted line is a guide to the eye indicating the value of the normalized spectra at $5\hbar\omega_D \sim 90$ meV, $\omega_D \approx 18$ meV being the Debye frequency of the films. As from where $\lambda$ has been estimated.

We observe the point contact spectrum scales at different values of $R_{\rm PC,0}$, indicating
1. the accurate estimation of the PC diameter, and
2. the quantity $\rho/d_{\rm PC}\ {\rm d}R_{\rm PC}/{\rm d}V$ to be corresponding to a physical entity intrinsic to the system, supporting the Eq. S27 For the film with $F = 0.33$, we observe the prominence of the peaks for certain values of point contact resistance. However, the background is equal in both cases, supporting the consistency of the background.

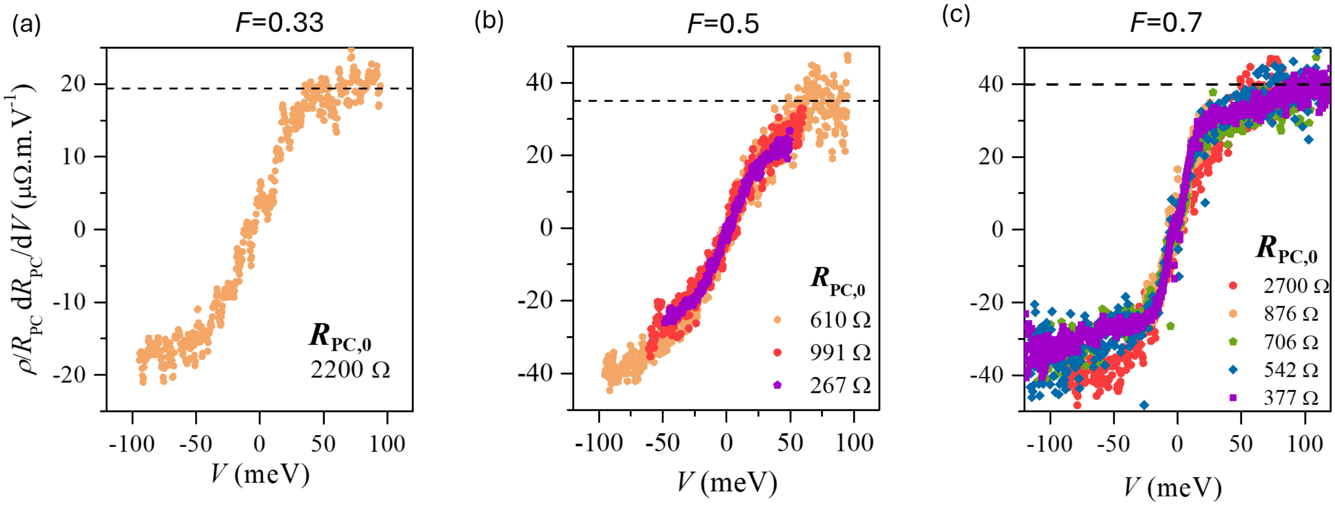


Fig. S17. Normalized point contact spectrum ($\rho/R_{\rm PC}\ {\rm d}R_{\rm PC}/{\rm d}V$) measured at different positions with different zero bias point contact resistances ($R_{\rm PC,0}$) for films with Ag-filling fraction $F = 0.33, 0.5, 0.7$ at temperatures $T \sim 6-8$ K.

3. Comment on any other $T$-dependent scattering mechanisms: We observe from Fig. 2c of the main manuscript and Fig. S17 that the background value of the PCS spectrum starts saturating at energy scales of $\sim 20$ meV, which corresponds to the acoustic phonon modes in Au. This energy scale is independent of the Ag fraction and is spatially homogeneous, as can be inferred from the scaling of the normalized point contact spectrum at different positions. These observations exclude any disorder-induced inelastic scattering that is generally stochastic in nature. We also exclude the possibility of electron-electron interaction to be the dominant inelastic scatterer in the experimentally measured region of bias $\pm 100$ meV since electron-electron interaction in metals is generally insignificant at these temperatures ($T \sim 6-8$ K).

## C. Measurement of Au film

Fig. S18(a) shows the measured bias dependences of the point contact resistance ($R_{\rm pc} = {\rm d}V/{\rm d}I$) and that of the derivative of $R_{\rm pc}$ (${\rm d}R_{\rm pc}/{\rm d}V$) at the zero-bias point contact resistance of $\sim 22\ \Omega$.

The Migdal Eliashberg function [55] $\mathcal{G}(\epsilon = eV) \approx \pi n e d_{\rm pc}\hbar/16m = \beta {\rm d}R_{\rm pc}/{\rm d}V$ is computed from the measured ${\rm d}R_{\rm pc}/{\rm d}V$ following Eq. [S14]. $d_{\rm pc}$, evaluated from Eq. [S8], using $v_{\rm F} = 1.43 \times 10^6$ m.s$^{-1}$, is $\sim 16$ nm for $R_{\rm pc} \sim 22\ \Omega$. Since the mean free path of Au ($l_{\rm Au}$) $\sim 17$ nm (using $\rho \sim 2.5 \times 10^{-8}\ \Omega$.m as the Drude resistivity), the point contact is in the ballistic regime. Hence, the spectrum shows a distinct peak around 5 meV, corresponding to the longitudinal acoustic (LA) peak of Au [55]. The transverse acoustic (TA) peak is not clear, probably due to the polycrystallinity of the thin film.

Fig. S18(b) shows the evaluation of $\lambda$ by integrating $\beta {\rm d}R_{\rm pc}/{\rm d}V = \mathcal{G}(\epsilon = eV)$ following Eq. [S15]. However, in the presence of a non-zero background in ${\rm d}R_{\rm pc}/{\rm d}V$ due to the non-ideal nature of the contact, $\lambda$ does not saturate. Since $\hbar\omega_{\rm D} \approx 18$ meV ($\omega_{\rm D}$ is the Debye frequency of Au), the integrated value at 18 meV (represented by the dotted line) is taken as $\lambda = 0.18$.

The presence of a non-zero background indicates the inhomogeneous nature of the point contact and the fact that $\beta {\rm d}R_{\rm pc}/{\rm d}V$ is overestimating $\mathcal{G}(\epsilon)$, thereby overestimating $\lambda$. Measurements were performed at a lower $R_{\rm pc} \sim 10\ \Omega$.

Fig. S18(c) shows the point contact spectrum ($dR_{pc}/dV$) normalized with $d_{pc}$ for different values of $R_{pc}$. The spectrum at $R_{pc} = 9.7\ \Omega$, shows smearing of the phonon peaks and higher background as compared to that of $R_{pc} \sim 22\ \Omega$. This is because of the point contact approaching a 'diffusive' nature, which is supported by the estimation (from Eq. [S8]) $d_{pc}(R_{pc} \approx 10\ \Omega) \approx 10$ nm $\lesssim l_{Au}$. However, both the traces overlap with approximately equal values of the background in Fig. S18(c), which gives $\lambda \approx 0.16$ following Eq. [1] of the main manuscript, indicating agreement within 10% with the previous estimates. The slight mismatch between the two traces could be caused by the uncertainty in the geometry of the point contact, which determines the exact Sharvin and Maxwell resistances and, consequently, the value of $d_{pc}$.

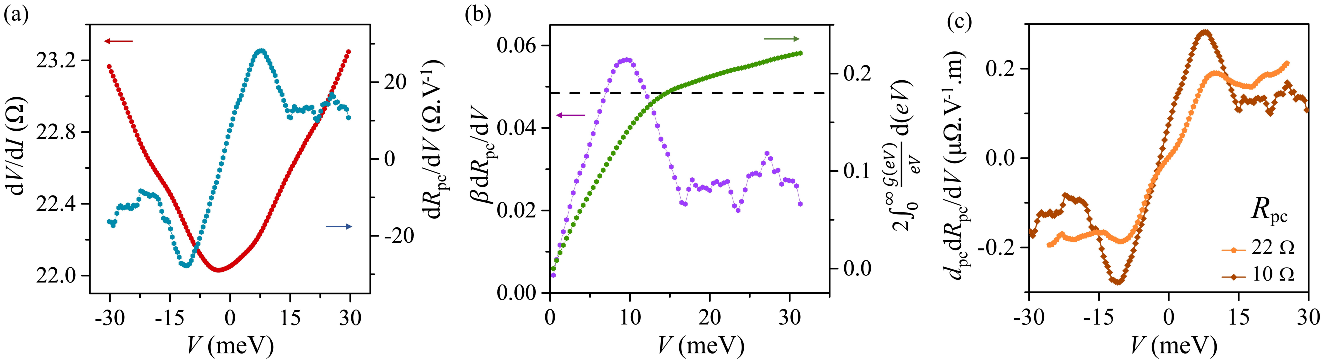


Fig. S18. **Point contact spectrum for Au film:** (a) Bias ($V$) dependences of point contact resistance $R_{pc} = dV/dI$ and derivative of $R_{pc}$, ($dR_{pc}/dV$). (b) Estimation of $\lambda$ by integrating $\beta dR_{pc}/dV = g(\epsilon = eV)$. The dashed line indicates that the integrated value of $[2\mathcal{G}(eV)/eV]$ at $eV \approx 18$ meV is assumed as $\lambda$. (c) $dR_{pc}/dV$ normalized with $d_{pc}$ for different point contact resistances.

### D. Measurement on AuNP film

Fig. S19 shows the point contact spectrum measured in a film of Au nanoparticles. The film was prepared following the protocol discussed in Sec. III above. Fig. S19(a) and (b) show the bias ($V$) dependences of the measured point contact resistance $\mathrm{d}V/\mathrm{d}I = R_{\mathrm{pc}}$ and derivative of $R_{\mathrm{pc}}$ ($\mathrm{d}R_{\mathrm{pc}}/\mathrm{d}V$) at two different values of $R_{\mathrm{pc}}$. $d_{\mathrm{pc}}$ is evaluated from Eq. [S8] with the resistivity of the film measured as $0.12\ \mu\Omega$.m. This gives a Drude mean free path of $\sim 3$ nm, whereas $d_{\mathrm{pc}}$ estimated from Eq. [S8] is typically $\sim 4-6$ nm, indicating that both the Sharvin and Maxwell contributions can have significant contributions to the point contact resistance. In such cases, the exact ballistic Sharvin resistance is dependent on the geometry of the point contact. To evaluate this experimentally, we have used a prefactor ($\simeq 0.35$) to the Sharvin resistance such that the normalized PC spectrum, $d_{\mathrm{pc}}\mathrm{d}R_{\mathrm{pc}}/\mathrm{d}V$ taken at two distinct positions overlap each other (shown in Fig. S19c).

$\lambda$ is calculated from the background of $d_{\mathrm{pc}}\mathrm{d}R_{\mathrm{pc}}/\mathrm{d}V$ as $\approx 0.45$ following Eq. [1] of the main manuscript. This is almost three times higher than that of Au film ($\sim 0.18$), which is consistent with earlier reports on the increase in electron-phonon coupling in Au/Ag nanoparticles [43] and is attributed to the surface scattering-mediated enhancement of electron-phonon coupling in nanoparticles.

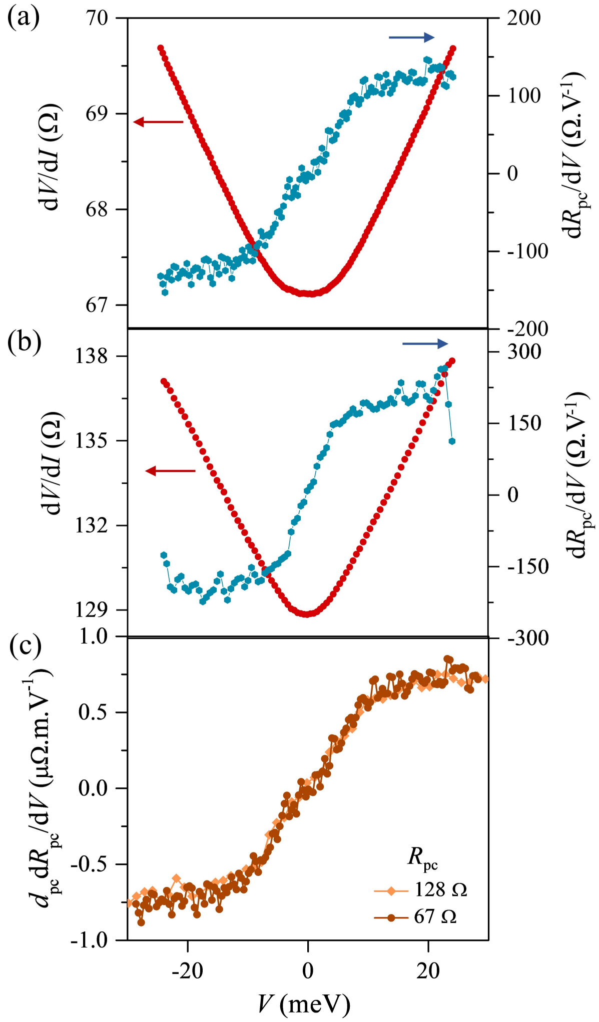


Fig. S19. **Point contact spectrum for AuNP film:** (a) and (b) show the bias ($V$) dependences of point contact resistance $R_{\mathrm{pc}} = \mathrm{d}V/\mathrm{d}I$ and derivative of $R_{\mathrm{pc}}$ ($\mathrm{d}R_{\mathrm{pc}}/\mathrm{d}V$) for different values of $R_{\mathrm{pc}}$. (c) $\mathrm{d}R_{\mathrm{pc}}/\mathrm{d}V$ normalized with $d_{\mathrm{pc}}$ for different point contact resistances.

## VIII. COMPILATION OF ELECTRON-PHONON COUPLING STRENGTH AND RESISTIVITY IN DIFFERENT MATERIALS

| Material | $\lambda$ | $\rho_{300\text{ K}}$ ($\mu\Omega$.m) | $\rho_{\text{MIR}}$ ($\mu\Omega$.m) | References |
|---|---|---|---|---|
| Ca | 0.05 | 0.03 | 9.2 | [41, 56] |
| K | 0.11 | 0.07 | 16.6 | [41, 56] |
| Ag | 0.12 | 0.016 | 9.4 | [41, 56] |
| Cu | 0.13 | 0.017 | 8.06 | [41, 56] |
| Na | 0.14 | 0.04 | 13.5 | [41, 56] |
| Rb | 0.15 | 0.115 | 16.9 | [41, 56] |
| Au | 0.15 | 0.022 | 8.8 | [41, 56] |
| Cs | 0.16 | 0.188 | 19.2 | [41, 56] |
| Mg | 0.2 | 0.044 | 9.3 | [41, 56] |
| Li | 0.35 | 0.092 | 11.36 | [41, 56] |
| Pd | 0.47 | 0.105 | 9.06 | [41, 56] |
| Sc | 0.51 | 0.505 | 8.68 | [41, 56] |
| Y | 0.62 | 0.55 | 9.09 | [41, 56] |
| Pt | 0.66 | 0.105 | 6.08 | [41, 56] |
| Al | 0.38 | 0.026 | 6.95 | [41, 56] |
| Th | 0.56 | 0.18 | 0.1065 | [41, 56] |
| Re | 0.46 | 0.172 | 0.145 | [41, 56] |
| In | 0.69 | 0.088 | 2.76 | [41, 56] |
| Hg | 1 | 0.985 | 5.27 | [41, 56] |
| Ga | 0.4 | 0.136 | 16.97 | [41, 56] |
| Sn | 0.6 | 0.1 | 2.78 | [41, 56] |
| Bi | 2.46 | 1.25 | 230.38 | [41, 56, 57] |
| $MgB_2$ | 0.8 | 0.1 | 5.84 | [41, 58] |
| $Bi_2Sr_2CaCu_2O_8$ | 3.34 | 3 | 24.45 | [59, 60] |
| $YBa_2Cu_3O_7$ | 0.25 | 2 | 13.47 | [61–64] |
| $La_{1.85}Sr_0.15CuO_4$ | 0.5 | 4 | 12.5 | [61, 63–66] |
| $ReO_3$ | 1.35 | 0.089 | 14.74 | [41, 56] |
| NbO | 1.4 | 0.21 | 13.8 | [67, 68] |

**Table S2**: Electron-phonon coupling constant ($\lambda$), room temperature resistivity ($\rho_{300\text{ K}}$), Mott-Ioffe-Regel resistivity ($\rho_{\text{MIR}}$) [48] for different materials that have been plotted in Fig. 3(b) of the main manuscript.

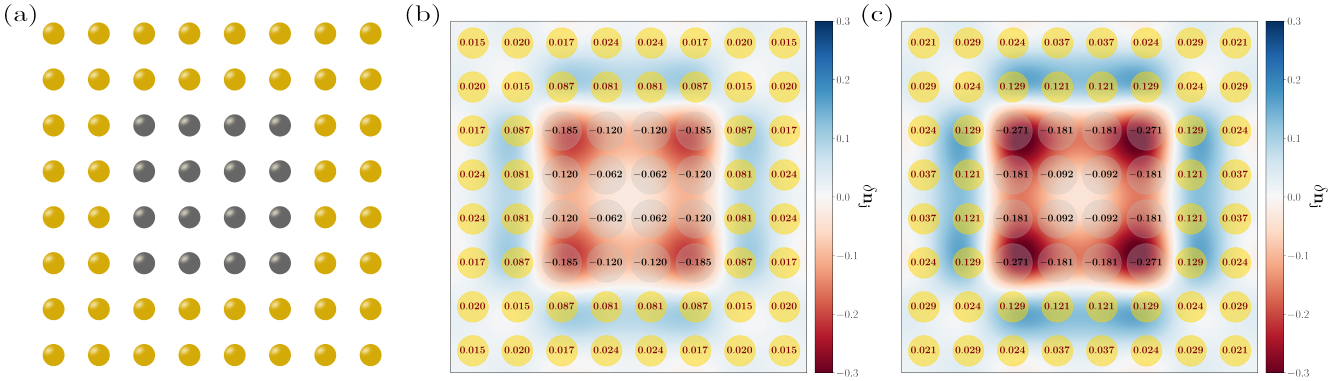


Fig. S20. **Theoretical calculations of the charge transfer in a model 2D "Au@Ag" super-lattice:** (a) Two-dimensional lattice used for the calculations: A square lattice, with a super-cell of dimension $(8 \times 8)$, with a $(4 \times 4)$ matrix of"Ag" atoms (silver dots) embedded within a matrix of "Au" atoms (golden dots) is considered in our model. The charge transfer in this lattice for $\epsilon_0 = (\epsilon_j^{\rm Ag} - \epsilon_j^{\rm Au}) = 1.0$ eV and $\epsilon_0 = 1.5$ eV shown in terms of the excess electron occupancy $\delta n_j$ at each of the Au and the Ag, lattice sites are plotted in (b) and (c). The average electron occupancy on Au atoms ($\langle \delta n_{\rm Au} \rangle$) is 0.03 and 0.04, respectively, in each of the cases, indicating that the charge transfer increases with increased $\epsilon_0$.

## IX. THEORETICAL MODEL FOR THE CALCULATION OF THE ELECTRON-PHONON COUPLING CONSTANT

To explore theoretically the properties of the engineered bimetallic nano-structured superlattices, we have used a simple two-dimensional lattice model. We consider a periodic square array of "Ag" atoms embedded within a matrix of "Au" atoms as shown in Fig. S20(a). The electronic Hamiltonian is modelled by assuming that the low-energy excitations involve only the s-like conduction bands of Au and Ag. The operators $\boldsymbol{c}_{j\mathbf{R}\sigma}$ and $\boldsymbol{c}^{\dagger}_{j\mathbf{R}\sigma}$ destroy and create electrons at the maximally localized Wannier orbitals localized at the Au/Ag sites labelled by $j\mathbf{R}$ where $j$ is the basis index and $\mathbf{R}$ a superlattice vector. The hopping amplitude between the Wannier orbitals at a pair of sites is denoted as $t_{j\mathbf{R};j'\mathbf{R}'}$ and is assumed to be only a function of the distance between the two sites, $d_{j\mathbf{R};j'\mathbf{R}'} = |(\mathbf{R} + \mathbf{d_j}) - (\mathbf{R}' + \mathbf{d_{j'}})|$. We model it as an exponentially decaying form $t_{j\mathbf{R};j'\mathbf{R}'} = t_0 \exp[-(d_{j\mathbf{R};j'\mathbf{R}'} - d_1)/\xi_0]$, where $d_1$ is the first neighbor distance of $\approx 0.41$ nm. We include Coulomb interactions in our model with the onsite Coulomb energy being denoted by $U_0$. The long-range Coulomb interaction between the electrons localized at sites $j\mathbf{R}$ and $j'\mathbf{R}'$ is assumed to scale inversely as the distance between the sites: $V(d_{j\mathbf{R};j'\mathbf{R}'}) = V_0/d_{j\mathbf{R};j'\mathbf{R}'}$. The local on-site energy $\epsilon_j$, independent of $\mathbf{R}$, is assumed to be different for the Au and Ag sites.

The total electronic Hamiltonian then can be written in terms of its contribution from each of these ingredients as

$$\boldsymbol{H}_{el} = \boldsymbol{H}_{hop} + \boldsymbol{H}_{ce} + E_{core} \tag{S29}$$

$$\boldsymbol{H}_{hop} = -\sum_{j,\mathbf{R};j',\mathbf{R}'}{}' \mathrm{t}_{j\mathbf{R};j'\mathbf{R}'} \sum_{\sigma} \boldsymbol{c}^{\dagger}_{j\mathbf{R}\sigma} \boldsymbol{c}_{j'\mathbf{R}'\sigma} + \sum_{j,\mathbf{R}} (\epsilon_j - \mu)\,(\boldsymbol{n}_{j\mathbf{R}} - 1) \tag{S30}$$

$$\boldsymbol{H}_{ce} = U_0 \sum_{j,\mathbf{R}} (\boldsymbol{n}_{j\mathbf{R}\uparrow} - \frac{1}{2})(\boldsymbol{n}_{j\mathbf{R}\downarrow} - \frac{1}{2}) + \frac{V_0}{2} \sum_{j,\mathbf{R};j',\mathbf{R}'}{}' \frac{1}{d_{j\mathbf{R};j'\mathbf{R}'}} (\boldsymbol{n}_{j\mathbf{R}} - 1)\,(\boldsymbol{n}_{j'\mathbf{R}'} - 1) \tag{S31}$$

Here $\boldsymbol{n}_{j\mathbf{R}\sigma} = \boldsymbol{c}^{\dagger}_{j\mathbf{R}\sigma}\boldsymbol{c}_{j\mathbf{R}\sigma}$ and $\boldsymbol{n}_{j\mathbf{R}} = \sum_{\sigma} \boldsymbol{n}_{j\mathbf{R}\sigma}$ are the spin resolved and the total number operator at the sites. The chemical potential $\mu$ is determined by the 'half-filled' condition, that the total number of electrons is exactly one per atomic site. This Hamiltonian is solved within restricted Hartree approximation and the excess electron occupancy at each atomic site $\delta n_j = \langle \boldsymbol{n}_j - 1 \rangle$ is obtained.

The transfer of charge is influenced by the difference in onsite potentials between the Ag and Au sites, $\epsilon_0 = (\epsilon_j^{\rm Ag} - \epsilon_j^{\rm Au})$. However, the calculated charge transfer, quantified by the average electron occupancy of Au atoms ($\langle \delta n_{\rm Au} \rangle \sim 0.03$), for $\epsilon_0 = 1$ eV, reflecting the difference in work functions between bulk Au and Ag, is $\langle \delta n_{\rm Au} \rangle \sim 0.03$, which is lower than what is observed in the XPS data for the Au@Ag NH (For $F = 0.5$, an average electron doping of the Au atoms by as much as $\sim 0.6 - 0.7$ is estimated from XPS measurements, See Fig. 3d of main manuscript and Supplementary Information Section II.B). The heightened charge transfer observed experimentally in the real system, in contrast to

the theoretical model, could be due to the reaction pathway. This pathway involves the reduction of silver salt to silver nanoparticles (AgNPs), followed by the reduction of gold on the AgNP surface, resulting in electron deficiency on the AgNP surface. The positively charged Ag interface impedes the reduction of Ag atoms ($Ag^0$ to $Ag^{+1}$) by $AuCl_4^{-1}$, i.e., the galvanic replacement by Au atoms, thereby preserving the stability of the Ag/Au interface. An additional mechanism that could contribute to large charge transfers is the formation of local bi-polarons due to the large local electron-phonon interactions that can result from our model (see below). We note that the bi-polaronic mechanism would also provide an explanation for the activated form of the parallel channel for conduction discussed previously, with the activation energy being given by the difference, arising from coulomb interactions, between the binding energies of a bi-polaron and a polaron. However, neither of above mechanisms are presently included in our calculations. To mimic experimental conditions, as shown in Fig. 3(d) in the main manuscript, we varied $\epsilon_0$ over a reasonable range to obtain much larger ranges of $\delta n$ seen in the experiments. Theoretical predictions of increasing charge transfer for different $\epsilon_0$ values are illustrated in Fig. S20(b) and (c).
For computing the phonons, the dynamical matrix is constructed as

$$D_{jj'}^{\alpha\alpha'}(\mathbf{q}) = \frac{1}{\sqrt{M_j M_j'}} \sum_{\mathbf{R}'} \left[ e^{i\mathbf{q}\cdot d_{j\mathbf{R};j'\mathbf{R}'}} \kappa_{jj'}^{\alpha\alpha'}(\mathbf{R}-\mathbf{R}') \right] \tag{S32}$$

Here, $\kappa_{jj'}^{\alpha\alpha'}$ is the force constant tensor, and $\alpha, \alpha'$ denotes the appropriate Cartesian components of the vectors and the tensors involved, and $M_j$ is the Mass of the Ag or Au atom depending on which of the atoms occupies the site $j$. For the purposes of the calculations presented in this paper, we model the force constant tensor in terms of just the nearest neighbour and the next-nearest neighbour bond stretching spring constants, which we choose so as to roughly reproduce the phonon spectra of pure Au and Ag [69]. The phonon frequencies ($\omega_{\mathbf{q}s}$) and the polarization vector components $\eta_{j;\mathbf{q}s}^{\alpha}$ are determined as the

$$\sum_{\alpha' j'} D_{jj'}^{\alpha\alpha'}(\mathbf{q}) \eta_{j';\mathbf{q}s}^{\alpha'} = \omega_{\mathbf{q}s}^2 \eta_{j;\mathbf{q}s}^{\alpha} \tag{S33}$$

The electron-phonon coupling matrix element, $g_{m'ms}(\mathbf{k},\mathbf{q})$, can be computed as the sum of two distinct contributions, one arising from the hopping part of the hamiltonian

$$\mathfrak{g}_{m'm;s}^{(hop)}(\mathbf{k},\mathbf{q}) = \sum_{j,j',\mathbf{R}'}{}' \frac{\mathfrak{t}(d_{j\mathbf{R};j'\mathbf{R}'})}{\xi_0} \left( \varphi_{j';(\mathbf{k}+\mathbf{q})m'}^{*} \varphi_{j;\mathbf{k}m} e^{i(\mathbf{k}+\mathbf{q})\cdot\mathbf{d}_{j\mathbf{R};j'\mathbf{R}'}} \right) \Upsilon_{jj';\mathbf{q}s}(\mathbf{R}-\mathbf{R}'), \tag{S34}$$

and the other from the Coulomb interaction part

$$\mathfrak{g}_{m'm;s}^{(ce)}(\mathbf{k},\mathbf{q}) = -\sum_{j,j',\mathbf{R}'}{}' \frac{V_0 \delta n_{j'}}{d_{j\mathbf{R};j'\mathbf{R}'}^2} \left( \varphi_{j;(\mathbf{k}+\mathbf{q})m'}^{*} \varphi_{j;\mathbf{k}m} \right) \Upsilon_{jj';\mathbf{q}s}(\mathbf{R}-\mathbf{R}'); \tag{S35}$$

where

$$\Upsilon_{jj';\mathbf{q}s}(\mathbf{R}-\mathbf{R}') \equiv \sqrt{\frac{\hbar}{2\omega_{\mathbf{q}s}}} \sum_{\alpha} \frac{d_{j\mathbf{R};j'\mathbf{R}'}^{\alpha}}{d_{j\mathbf{R};j'\mathbf{R}'}} \left[ \frac{\eta_{j;\mathbf{q}s}^{\alpha}}{\sqrt{M_j}} - \frac{\eta_{j';\mathbf{q}s}^{\alpha}}{\sqrt{M_{j'}}} e^{-i\mathbf{q}\cdot\mathbf{d}_{j\mathbf{R};j'\mathbf{R}'}} \right]. \tag{S36}$$

Here, $\varphi_{j,\mathbf{k}m}$ is the components at site j of the electronic eigenvector for wave-vector $\mathbf{k}$ and band index m. We emphasize that the second contribution (eqn. S35) to the electron phonon coupling matrix element arises only in contexts when $\delta n$ is non-zero, as in the case of Ag@Au-NH, and would be absent in pure Au or Ag.
The Migdal-Eliashberg electron-phonon spectral function, $\alpha^2\mathcal{F}(\omega)$, is computed under the 'double-delta' approximation as

$$\alpha^2\mathcal{F}(\omega) \equiv \frac{1}{(\mathcal{L})^2\mathcal{D}(0)} \sum_{\mathbf{k},m,m'} \sum_{\mathbf{q},s} |\mathfrak{g}_{m'm;s}(\mathbf{k},\mathbf{q})|^2 \, \delta(\varepsilon_{\mathbf{k}m}) \, \delta(\varepsilon_{(\mathbf{k}+\mathbf{q})\,m'}) \, \delta(\hbar\omega - \hbar\omega_{\mathbf{q}s}). \tag{S37}$$

where

$$\mathcal{D}(0) = \frac{1}{\mathcal{L}} \sum_{\mathbf{k},m} \delta(\varepsilon_{\mathbf{k}m}) \tag{S38}$$

is the electronic Density of states (DOS) at the Fermi level. Using this, the electron-phonon coupling strength ($\lambda^{(\text{calc})}$) is obtained as

$$\lambda^{(\text{calc})} = 2\int_0^\infty d\omega \frac{\alpha^2\mathcal{F}(\omega)}{\omega} \tag{S39}$$

We note that, in regimes where the Coulomb interaction contribution (eqn. S35) to $\lambda^{(\text{calc})}$ dominates, it will rise rapidly with the magnitude of the charge transfer, as $(\delta n)^2$. This calculation of $\lambda^{(\text{calc})}$ does not include the change in effective mass. The details of the derivation of the above expressions as well as the calculations of the EPC including many more results will be presented elsewhere [70].

## X. STRESS SIMULATIONS

Vibrational eigenmodes of the Ag@Au nanohybrid are simulated using the Structural Mechanics Module in COMSOL. Fig. S21(a) shows the schematic of the geometry used for simulations. AgNPs of radii $\sim 1.5$ nm are embedded inside a larger Au sphere of radius $\sim 12.5$ nm. The nearest nanoparticles are positioned at equal distances from each other, thus following a cubic arrangement. The number of AgNPs inside the Au host is varied to tune the center-to-center distance between the AgNPs ($d_{\rm Ag}$). The parameters used for the simulations are consistent with a previous report [71] and are as follows: Density $\rho_{\rm d,Au} = 19300$ kg.m$^{-3}$, Young's Modulus $E_{\rm Au} = 79$ GPa, and Poisson's ratio $\nu_{\rm Au} = 0.42$ for Au, and $\rho_{\rm d,Ag} = 10500$ kg.m$^{-3}$, $E_{\rm Ag} = 75.1$ GPa, and $\nu_{\rm Ag} = 0.5$ for Ag. Stress and displacement fields can be estimated for each eigenfrequency for a given value of $d_{\rm Ag}$. The volume average of the hydrostatic pressure developed inside the nanoparticle is taken as a measure of stress. If the net magnitude is positive, then the stress is compressive, whereas a negative value indicates tensile stress. Fig. S21(b) shows the generation of a high magnitude of tensile stress inside the hybrid system for lower values of $d_{\rm Ag}$. For a quantitative comparison, tensile stress magnitude at a fixed eigenfrequency $\sim 77$ Hz is plotted as a function of $d_{\rm Ag}$ in Fig. S21(c). We observed an increase in stress by 3 orders of magnitude with higher Ag fractions, equivalent to lower $d_{\rm Ag}$. In many materials like noble metals and $MgB_2$, the electron-phonon coupling constant ($\lambda$) has been shown to decrease with compressive stress and increase with tensile stress [72–74]. However, the change in $\lambda$ in Au/Ag has been reported theoretically [72] to be within 50% even with 10 orders of magnitude more pressure ($\sim 100$ GPa). Though the existing reports only predict the effect of compressive strain, we can use the relative change as a typical estimate for change in $\lambda$ with tensile strain. Hence, the enhancement of $\lambda$ by about 100 times ($\sim 10^4$ %) in our experiment is unlikely to be explained only by the intrinsic mechanical stress in our embedded system of Ag@Au.

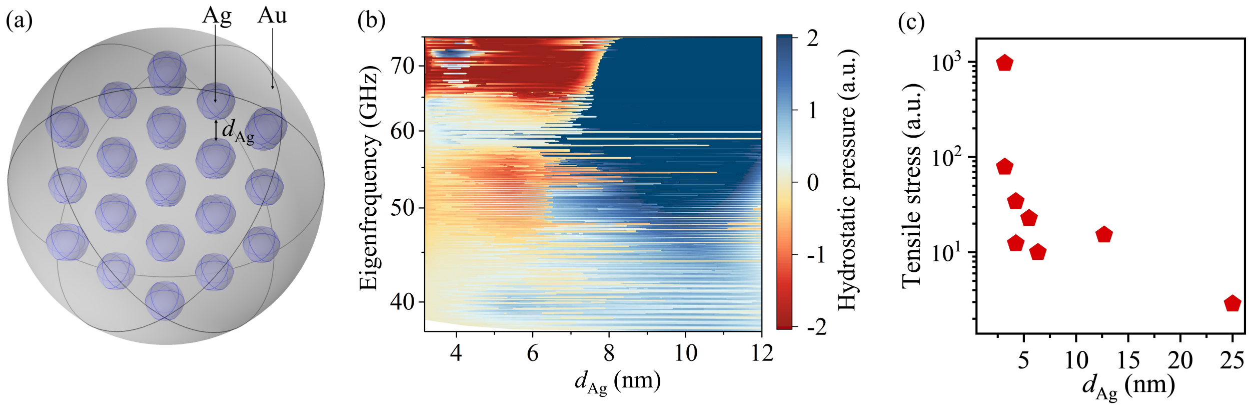


Fig. S21. **Vibrational eigenmodes and stress in Ag@Au hybrid**. (a) Schematic of the geometry used in COMSOL. Purple spheres represent Ag nanoparticles embedded inside a larger Au nanoparticle represented by the grey sphere. (b) Distribution of the hydrostatic pressure generated inside the hybrid system as a function of the centre-to-centre distance between the AgNPs ($d_{\rm Ag}$) at different eigenfrequencies. (c) Tensile stress at a frequency $\sim 77$ Hz as function of ($d_{\rm Ag}$).

## REFERENCES


[1] Maji, T. K.; Kumbhakar, S.; Tongbram, B.; Sai, T. P.; Islam, S.; Mahapatra, P. S.; Pandey, A.; Ghosh, A. Electrical Resistance in a Composite of Ultra-Small Silver Nanoparticles Embedded in Gold Nanostructures: Implications for Interface-Enabled Functionality. *ACS Appl. Electron. Mater.* **2023**, *5*, 2893–2901.

[2] Agnihotri, S.; Mukherji, S.; Mukherji, S. Size-controlled silver nanoparticles synthesized over the range 5–100 nm using the same protocol and their antibacterial efficacy. *Rsc Advances* **2014**, *4*, 3974–3983.

[3] Zhao, C.; Zhong, G.; Kim, D.-E.; Liu, J.; Liu, X. A portable lab-on-a-chip system for gold-nanoparticle-based colorimetric detection of metal ions in water. *Biomicrofluidics* **2014**, *8*, 052107.

[4] Shibata, T.; Bunker, B. A.; Zhang, Z.; Meisel, D.; Vardeman, C. F.; Gezelter, J. D. Size-dependent spontaneous alloying of Au- Ag nanoparticles. *Journal of the American Chemical Society* **2002**, *124*, 11989–11996.

[5] Krishnamurthy, S.; Esterle, A.; Sharma, N. C.; Sahi, S. V. Yucca-derived synthesis of gold nanomaterial and their catalytic potential. *Nanoscale research letters* **2014**, *9*, 1–9.

[6] Sakthisabarimoorthi, A.; Martin Britto Dhas, S.; Jose, M. Preparation of composite Ag@ Au core–shell nanoparticles and their linear and nonlinear optical properties. *Journal of Materials Science: Materials in Electronics* **2019**, *30*, 1677–1685.

[7] Sylvestre, J.-P.; Poulin, S.; Kabashin, A. V.; Sacher, E.; Meunier, M.; Luong, J. H. Surface chemistry of gold nanoparticles produced by laser ablation in aqueous media. *J. Phys. Chem. B* **2004**, *108*, 16864–16869.

[8] Lindberg, B. J.; Hamrin, K.; Johansson, G.; Gelius, U.; Fahlman, A.; Nordling, C.; Siegbahn, K. Molecular Spectroscopy by Means of ESCA II. Sulfur compounds. Correlation of electron binding energy with structure. *Physica Scripta* **1970**, *1*, 286.

[9] Grönbeck, H.; Klacar, S.; Martin, N. M.; Hellman, A.; Lundgren, E.; Andersen, J. N. Mechanism for reversed photoemission core-level shifts of oxidized Ag. *Phys. Rev. B* **2012**, *85*, 115445.

[10] Johansson, B.; Mårtensson, N. Core-level binding-energy shifts for the metallic elements. *Phys. Rev. B* **1980**, *21*, 4427–4457.

[11] Andersen, J. N.; Hennig, D.; Lundgren, E.; Methfessel, M.; Nyholm, R.; Scheffler, M. Surface core-level shifts of some 4d-metal single-crystal surfaces: Experiments and ab initio calculations. *Phys. Rev. B* **1994**, *50*, 17525–17533.

[12] Weinert, M.; Watson, R. E. Core-level shifts in bulk alloys and surface adlayers. *Phys. Rev. B* **1995**, *51*, 17168–17180.

[13] Abrikosov, I. A.; Olovsson, W.; Johansson, B. Valence-Band Hybridization and Core Level Shifts in Random Ag-Pd Alloys. *Phys. Rev. Lett.* **2001**, *87*, 176403.

[14] Steiner, P.; Hüfner, S. Thermochemical data of alloys from photoelectron spectroscopy. *Acta Metallurgica* **1981**, *29*, 1885–1898.

[15] Tal, A. A.; Olovsson, W.; Abrikosov, I. A. Origin of the core-level binding energy shifts in Au nanoclusters. *Phys. Rev. B* **2017**, *95*, 245402.

[16] Howard, A.; Clark, D.; Mitchell, C.; Egdell, R.; Dhanak, V. Initial and final state effects in photoemission from Au nanoclusters on TiO2(110). *Surface Science* **2002**, *518*, 210–224.

[17] Wertheim, G. K.; DiCenzo, S. B.; Youngquist, S. E. Unit Charge on Supported Gold Clusters in Photoemission Final State. *Phys. Rev. Lett.* **1983**, *51*, 2310–2313.

[18] Dalacu, D.; Klemberg-Sapieha, J. E.; Martinu, L. Substrate and morphology effects on photoemission from core-levels in gold clusters. *Surface Science* **2001**, *472*, 33–40.

[19] Casaletto, M. P.; Longo, A.; Martorana, A.; Prestianni, A.; Venezia, A. M. XPS study of supported gold catalysts: the role of Au0 and Au+ species as active sites. *Surface and Interface Analysis* **2006**, *38*, 215–218.

[20] Venezia, A. M.; Pantaleo, G.; Longo, A.; Di Carlo, G.; Casaletto, M. P.; Liotta, F. L.; Deganello, G. Relationship between structure and CO oxidation activity of ceria-supported gold catalysts. *The Journal of Physical Chemistry B* **2005**, *109*, 2821–2827.

[21] Park, E. D.; Lee, J. S. Effects of pretreatment conditions on CO oxidation over supported Au catalysts. *Journal of Catalysis* **1999**, *186*, 1–11.

[22] Klyushin, A. Y.; Rocha, T. C.; Hävecker, M.; Knop-Gericke, A.; Schlögl, R. A near ambient pressure XPS study of Au oxidation. *Physical Chemistry Chemical Physics* **2014**, *16*, 7881–7886.

[23] Müllegger, S.; Schöfberger, W.; Rashidi, M.; Lengauer, T.; Klappenberger, F.; Diller, K.; Kara, K.; Barth, J. V.; Rauls, E.; Schmidt, W. G.; Koch, R. Preserving Charge and Oxidation State of Au(III) Ions in an Agent-Functionalized Nanocrystal Model System. *ACS Nano* **2011**, *5*, 6480–6486, PMID: 21736315.

[24] Kalinkin, A.; Smirnov, M. Y.; Bukhtiyarov, A.; Bukhtiyarov, V. XPS study of gold oxidation with nitrogen dioxide in model Au/C samples. *Kinetics and Catalysis* **2015**, *56*, 796–800.

[25] Yakimchuk, D. V.; Bundyukova, V. D.; Ustarroz, J.; Terryn, H.; Baert, K.; Kozlovskiy, A. L.; Zdorovets, M. V.; Khubezhov, S. A.; Trukhanov, A. V.; Trukhanov, S. V.; others Morphology and microstructure evolution of gold nanostructures in the limited volume porous matrices. *Sensors* **2020**, *20*, 4397.

[26] Lanza, G.; Martinez Jimenez, M. J.; Alvarez, F.; Perez-Taborda, J. A.; Avila, A. Valence State Tuning of Gold Nanoparticles in the Dewetting Process: An X-ray Photoelectron Spectroscopy Study. *ACS Omega* **2022**, *7*, 34521–34527.

[27] Pramanik, G.; Humpolickova, J.; Valenta, J.; Kundu, P.; Bals, S.; Bour, P.; Dracinsky, M.; Cigler, P. Gold nanoclusters with bright near-infrared photoluminescence. *Nanoscale* **2018**, *10*, 3792–3798.

[28] Grönbeck, H. The bonding in thiolate protected gold nanoparticles from Au4f photoemission core level shifts. *Nanoscale* **2012**, *4*, 4178–4182.

[29] Yadav, V.; Jeong, S.; Ye, X.; Li, C. W. Surface-Limited Galvanic Replacement Reactions of Pd, Pt, and Au onto Ag Core Nanoparticles through Redox Potential Tuning. *Chemistry of Materials* **2022**, *34*, 1897–1904.

[30] Gao, C.; Lu, Z.; Liu, Y.; Zhang, Q.; Chi, M.; Cheng, Q.; Yin, Y. Highly Stable Silver Nanoplates for Surface Plasmon Resonance Biosensing. *Angewandte Chemie International Edition* **2012**, *51*, 5629–5633.

[31] Philips'Gloeilampenfabrieken, O. A method of measuring specific resistivity and Hall effect of discs of arbitrary shape. *Philips Res. Rep* **1958**, *13*, 1–9.

[32] Qin, X. Y.; Zhang, W.; Zhang, L. D.; Jiang, L. D.; Liu, X. J.; Jin, D. Low-temperature resistance and its temperature dependence in nanostructured silver. *Phys. Rev. B* **1997**, *56*, 10596–10604.

[33] Moreira, H.; Yu, Q.; Nadal, B.; Bresson, B.; Rosticher, M.; Lequeux, N.; Zimmers, A.; Aubin, H. Electron Cotunneling Transport in Gold Nanocrystal Arrays. *Phys. Rev. Lett.* **2011**, *107*, 176803.

[34] Dugay, J.; Tan, R. P.; Ibrahim, M.; Garcia, C.; Carrey, J.; Lacroix, L.-M.; Fazzini, P.-F.; Viau, G.; Respaud, M. Charge transport and interdot coupling tuned by the tunnel barrier length in assemblies of nanoparticles surrounded by organic ligands. *Phys. Rev. B* **2014**, *89*, 041406.

[35] Herrmann, J.; Bray, D. J.; Müller, K.-H.; Wei, G.; Lindoy, L. F. Tuning the Coulomb charging energy in cross-linked nanoparticle films. *Phys. Rev. B* **2007**, *76*, 212201.

[36] Tran, T. B.; Beloborodov, I. S.; Hu, J.; Lin, X. M.; Rosenbaum, T. F.; Jaeger, H. M. Sequential tunneling and inelastic cotunneling in nanoparticle arrays. *Phys. Rev. B* **2008**, *78*, 075437.

[37] Liu, H.; Pourret, A.; Guyot-Sionnest, P. Mott and Efros-Shklovskii Variable Range Hopping in CdSe Quantum Dots Films. *ACS Nano* **2010**, *4*, 5211–5216.

[38] Duan, C.; Wang, Y.; Sun, J.; Guan, C.; Grunder, S.; Mayor, M.; Peng, L.; Liao, J. Controllability of the Coulomb charging energy in close-packed nanoparticle arrays. *Nanoscale* **2013**, *5*, 10258–10266.

[39] Simon, U. Charge transport in nanoparticle arrangements. *Adv. Mater.* **1998**, *10*, 1487–1492.

[40] Likovich, E. M.; Russell, K. J.; Petersen, E. W.; Narayanamurti, V. Weak localization and mobility in ZnO nanostructures. *Phys. Rev. B* **2009**, *80*, 245318.

[41] Allen, P. B. The electron-phonon coupling constant. *Tc* **2000**, *500*, 45.

[42] Ziman, J. *Principles of the Theory of Solids*; Cambridge University Press, 1972.

[43] Staechelin, Y. U.; Hoeing, D.; Schulz, F.; Lange, H. Size-Dependent Electron–Phonon Coupling in Monocrystalline Gold Nanoparticles. *ACS Photonics* **2021**, *8*, 752–757.

[44] Gunnarsson, O.; Calandra, M.; Han, J. Colloquium: Saturation of electrical resistivity. *Rev. of Modern Phys.* **2003**, *75*, 1085.

[45] Mikheev, E.; Hauser, A. J.; Himmetoglu, B.; Moreno, N. E.; Janotti, A.; de Walle, C. G. V.; Stemmer, S. Tuning bad metal and non-Fermi liquid behavior in a Mott material: Rare-earth nickelate thin films. *Science Advances* **2015**, *1*, e1500797.

[46] Werman, Y.; Berg, E. Mott-Ioffe-Regel limit and resistivity crossover in a tractable electron-phonon model. *Phys. Rev. B* **2016**, *93*, 075109.

[47] Werman, Y.; Kivelson, S. A.; Berg, E. Non-quasiparticle transport and resistivity saturation: a view from the large-N limit. *npj Quantum Mater.* **2017**, *2*, 7.

[48] Ioffe, A.; Regel, A. *Progress in semiconductors*; 1960; pp 237–291.

[49] Naidyuk, Y.; Yanson, I. *Point-Contact Spectroscopy*; Springer Series in Solid-State Sciences; Springer New York, 2019.

[50] Van Gelder, A. On the structure of the d2J/dV2 characteristics of point contacts between metals. *Solid State Communications* **1980**, *35*, 19–21.

[51] Kulik, I. Frequency dispersion caused in the conductivity of metal microcontacts by nonequilibrium-phonon relaxation. *Soviet Journal of Experimental and Theoretical Physics Letters* **1985**, *41*, 370.

[52] Kulik, I. O.; Ellialtiogammalu, R. *Quantum mesoscopic phenomena and mesoscopic devices in microelectronics*; Springer Science & Business Media, 2012; Vol. 559.

[53] Kulik, I. On the determination of $\alpha^2F(\omega)$ in metals by measuring I–V characteristics of "wide" (non-ballistic) point-contact junctions. *Physics Letters A* **1984**, *106*, 187–190.

[54] Baxter, D. V.; Richter, R.; Trudeau, M.; Cochrane, R.; Strom-Olsen, J. Fitting to magnetoresistance under weak localization in three dimensions. *Journal de Physique* **1989**, *50*, 1673–1688.

[55] Naidyuk, Y. G.; Yanson, I. K. *Point-contact spectroscopy*; Springer Science & Business Media, 2005; Vol. 145.

[56] Hurd, C. *The Hall Effect in Metals and Alloys*; The International Cryogenics Monograph Series; Springer US, 2012.

[57] Luo, N.; Miley, G. Kohler's rule and relaxation rates in high-Tc superconductors. *Physica C: Superconductivity* **2002**, *371*, 259–269.

[58] Kang, W.; Kim, H. J.; Kim, H.-J.; Choi, E.-M.; Kim, K. H.; Lee, H.; Lee, S.-I. Synthesis of c-axis-oriented MgB2 thin films and the Hall effect. *Superconductor Science and Technology* **2003**, *16*, 237.

[59] Forro, L.; Mandrus, D.; Kendziora, C.; Mihaly, L.; Reeder, R. Hall-effect measurements on superconducting and nonsuperconducting copper-oxide-based metals. *Phys. Rev. B* **1990**, *42*, 8704–8706.

[60] Ummarino, G. A.; Gonnelli, R. S. Breakdown of Migdal's theorem and intensity of electron-phonon coupling in high-$T_c$ superconductors. *Phys. Rev. B* **1997**, *56*, R14279–R14282.

[61] Gadermaier, C.; Alexandrov, A. S.; Kabanov, V. V.; Kusar, P.; Mertelj, T.; Yao, X.; Manzoni, C.; Brida, D.; Cerullo, G.; Mihailovic, D. Electron-Phonon Coupling in High-Temperature Cuprate Superconductors Determined from Electron Relaxation Rates. *Phys. Rev. Lett.* **2010**, *105*, 257001.

[62] Daou, R.; Doiron-Leyraud, N.; LeBoeuf, D.; Li, S.; Laliberté, F.; Cyr-Choiniere, O.; Jo, Y.; Balicas, L.; Yan, J.-Q.; Zhou, J.-S.; others Linear temperature dependence of resistivity and change in the Fermi surface at the pseudogap critical point of a high-T c superconductor. *Nature Physics* **2009**, *5*, 31–34.

[63] Göb, W.; Liebich, W.; Lang, W.; Puica, I.; Sobolewski, R.; Rössler, R.; Pedarnig, J. D.; Bäuerle, D. Double sign reversal of the vortex Hall effect in $YBa_2Cu_3O_{7-\delta}$ thin films in the strong pinning limit of low magnetic fields. *Phys. Rev. B* **2000**,

*62*, 9780–9783.

[64] Cyr-Choinière, O.; LeBoeuf, D.; Badoux, S.; Dufour-Beauséjour, S.; Bonn, D. A.; Hardy, W. N.; Liang, R.; Graf, D.; Doiron-Leyraud, N.; Taillefer, L. Sensitivity of $T_c$ to pressure and magnetic field in the cuprate superconductor $YBa_2Cu_3O_y$: Evidence of charge-order suppression by pressure. *Phys. Rev. B* **2018**, *98*, 064513.

[65] Alexandrov, A. S.; Zavaritsky, V. N.; Dzhumanov, S. Hall effect and resistivity in underdoped cuprates. *Phys. Rev. B* **2004**, *69*, 052505.

[66] Nikšić, G.; Kupčić, I.; Barišić, O.; Sunko, D.; Barišić, S. Multiband responses in high-T c cuprate superconductors. *Journal of Superconductivity and Novel Magnetism* **2014**, *27*, 969–975.

[67] Hulm, J.; Jones, C.; Hein, R.; Gibson, J. Superconductivity in the TiO and NbO systems. *Journal of Low Temperature Physics* **1972**, *7*, 291–307.

[68] Honig, J.; Wahnsiedler, W.; Eklund, P. Electrical properties of NbO in high magnetic fields. *Journal of Solid State Chemistry* **1973**, *6*, 203–212.

[69] Drexel, W. Lattice dynamics of silver. *Zeitschrift für Physik A Hadrons and nuclei* **1972**, *255*, 281–299.

[70] Mandal, S.; Soundararajan, S.; Jain, M.; Krishnamurthy, H. R. Possibilities for enhanced electron-phonon interactions and high-$T_c$ superconductivity in engineered bimetallic nano-structured superlattices. *arXiv* **2024**, *2408.15820*.

[71] Yu, S.; Zhang, J.; Tang, Y.; Ouyang, M. Engineering acoustic phonons and electron–phonon coupling by the nanoscale interface. *Nano Lett.* **2015**, *15*, 6282–6288.

[72] Giri, A.; Gaskins, J. T.; Li, L.; Wang, Y.-S.; Prezhdo, O. V.; Hopkins, P. E. First-principles determination of the ultrahigh electrical and thermal conductivity in free-electron metals via pressure tuning the electron-phonon coupling factor. *Phys. Rev. B* **2019**, *99*, 165139.

[73] Johansson, E.; Tasnádi, F.; Ektarawong, A.; Rosen, J.; Alling, B. The effect of strain and pressure on the electron-phonon coupling and superconductivity in MgB2—Benchmark of theoretical methodologies and outlook for nanostructure design. *Journal of Applied Physics* **2022**, *131*, 063902.

[74] Lanzillo, N. A.; Thomas, J. B.; Watson, B.; Washington, M.; Nayak, S. K. Pressure-enabled phonon engineering in metals. *Proc. Natl. Acad. Sci.* **2014**, *111*, 8712–8716.
